# Biomimetic 2D layered double hydroxide nanocomposites for hyperthermia-facilitated homologous targeting cancer photo-chemotherapy

**DOI:** 10.1186/s12951-021-01096-9

**Published:** 2021-10-30

**Authors:** Jingjing Wang, Luyao Sun, Jie Liu, Bing Sun, Li Li, Zhi Ping Xu

**Affiliations:** Australian Institute for Bioengineering and Nanotechnology, The University of Queensland, Brisbane, QLD 4072 Australia

**Keywords:** Biomimetic, Layered double hydroxide nanosheets, Homologous targeting, Hyperthermia, Immune escape, Photo-chemotherapy, colorectal carcinoma

## Abstract

**Background:**

Multi-modal therapy has attracted increasing attention as it provides enhanced effectiveness and potential stimulation of the immune community. However, low accumulation at the tumor sites and quick immune clearance of the anti-tumor agents are still insurmountable challenges. Hypothetically, cancer cell membrane (CCM) can homologously target the tumor whereas multi-modal therapy can complement the disadvantages of singular therapies. Meanwhile, moderate hyperthermia induced by photothermal therapy can boost the cellular uptake of therapeutic agents by cancer cells.

**Results:**

CCM-cloaked indocyanine green (ICG)-incorporated and abraxane (PTX-BSA)-loaded layered double hydroxide (LDH) nanosheets (LIPC NSs) were fabricated for target efficient photo-chemotherapy of colorectal carcinoma (CRC). The CCM-cloaked LDH delivery system showed efficient homologous targeting and cytotoxicity, which was further enhanced under laser irradiation to synergize CRC apoptosis. On the other hand, CCM-cloaking remarkably reduced the uptake of LDH NSs by HEK 293T cells and macrophages, implying mitigation of the side effects and the immune clearance, respectively. In vivo data further exhibited that LIPC NSs enhanced the drug accumulation in tumor tissues and significantly retarded tumor progression under laser irradiation at very low therapeutic doses (1.2 and 0.6 mg/kg of ICG and PTX-BSA), without observed side effects on other organs.

**Conclusions:**

This research has demonstrated that targeting delivery efficiency and immune-escaping ability of LIPC NSs are tremendously enhanced by CCM cloaking for efficient tumor accumulation and in situ generated hyperthermia boosts the uptake of LIPC NSs by cancer cells, a potential effective way to improve the multi-modal cancer therapy.

**Graphical Abstract:**

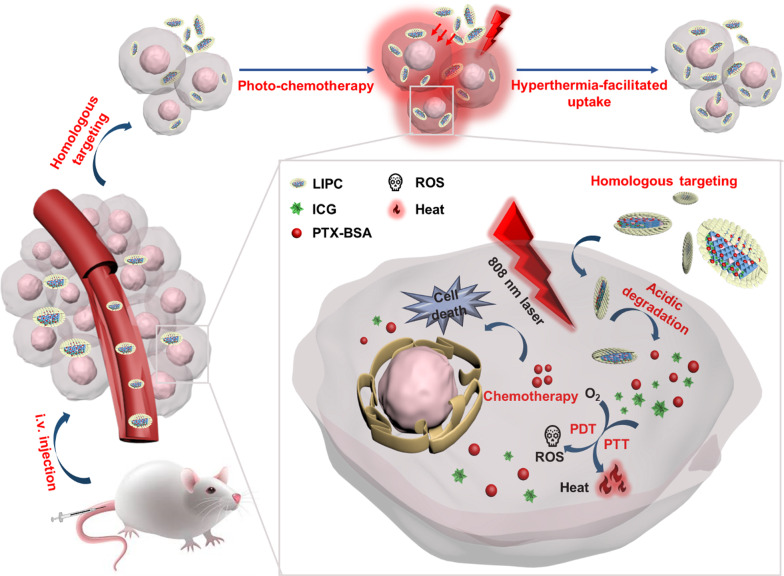

**Supplementary Information:**

The online version contains supplementary material available at 10.1186/s12951-021-01096-9.

## Background

Colorectal carcinoma (CRC) has become the second leading cause of cancer-related deaths, with two million cases diagnosed annually [1, 2]. Chemotherapy, as one of the primary treatments for CRC, has made great progress, while the five-year survival rate of stage IV patients is only 10% [3]. The limited effectiveness of chemotherapy is mainly attributed to off-target of the anti-tumor agents, drug resistance, and severe side effects. Recently, phototherapy, including photothermal (PTT) and photodynamic therapy (PDT), has been intensively exploited to suppress tumor growth through inducing hypothermia and reactive oxygen species (ROS) in the tumors using photosensitizers such as indocyanine green (ICG) under near-infrared (NIR) irradiation [4–6]. As a non-invasive and local therapy modality, phototherapy addresses drug resistance, increases the efficacy of chemotherapy, and reduces associated side effects. For example, the combination of phototherapy (such as ICG-based PTT) and chemotherapy (such as doxorubicin) delivered by polydopamine nanoparticles effectively improved breast cancer inhibition [7]. Encouragingly, the fabrication of targeted combination photo-chemotherapy would potentially provide a promising solution to enhance therapeutic outcomes of CRC.

On the other hand, cancer cell membrane (CCM), an active targeting motif and effective biomimetic material, has been coated on the nanoparticle surface for anti-tumor drug delivery to enhance therapeutic efficiency and minimize adverse effects [8, 9]. Owing to the adhesive proteins, homogenesis receptors and natural membrane structure, CCM-camouflaged nanoparticles possess the following specific properties: (1) homologous targeting [10]; (2) immune escape capability [11]; (3) improved colloidal stability; and (4) reduced leakage of anti-tumor agents [12]. Notably, hyperthermia at 43–45 °C was reported to accelerate the fluidity of CCM, resulting in elevated accumulation of anti-cancer agents [13]. Recent investigations have revealed that CCM-camouflaged nanoparticles including upconversion, semiconducting polymer, and ICG/poly(lactic-co-glycolic acid) nanoparticles achieved homologous targeting and enhanced phototherapy of breast cancers [12, 14, 15]. Interestingly, CCM-encapsulated porphyrin-loaded metal–organic framework with glucose oxidase/catalase incorporated has functioned as a cascade bioreactor for synergistic starvation and PDT of cancers [11]. Similarly, CCM-cloaked nanoparticles were also successfully utilized in combination therapies, such as PTT-chemotherapy, starvation-immunotherapy, and photo-immunotherapy [16–18]. Hence, CCM is a prosperous homologous targeting motif for delivering anti-tumor therapeutics and minimizing the off-target issue in cancer therapy.

Recently, our group has developed two-dimensional layered double hydroxide nanosheets (LDH NSs) as a platform for biomedical applications such as drug delivery, biosensing, tumor imaging, and therapy [19–21]. By incorporating Mn^2+^ or ^19^F magnetic resonance imaging (MRI) probe into LDH NSs, we have developed the pH-sensitive MRI imaging platforms for specific detection of cancers [22, 23]. On the other hand, LDH NSs improve the tumor accumulation for efficient chemotherapy and PTT [24–26]. Moreover, positively charged LDH NSs can electrostatically carry negatively charged drugs, siRNA, antigens, and CpG, for crossing the blood–brain barrier [27], gene delivery [28], and immunotherapy [19, 29].

**Scheme. 1:**
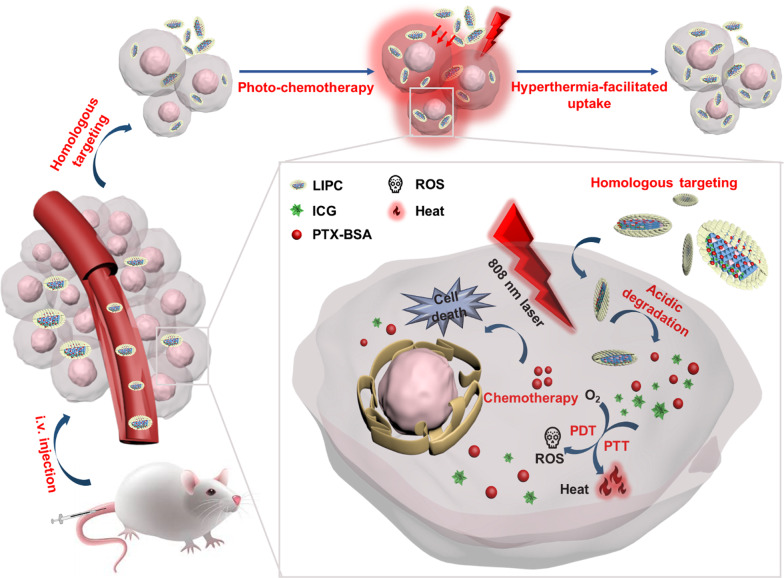
Schematic illustration of CCM-cloaked LDH NSs for hyperthermia-facilitated homologous targeting photo-chemotherapy

Herein, CCM-cloaked LDH nanomedicine was developed for homologous targeting hyperthermia-facilitated photo-chemotherapy by co-delivering ICG and Abraxane (PTX-BSA) (Scheme 1). The decoration of CCM not only endows LDH NSs with homologous targeting and immune-escaping ability, but also improves the colloidal stability during blood circulation, thereby benefiting the accumulation in the tumor tissues. Upon LDH NS accumulation in tumors and subsequent NIR irradiation, cancer cell growth is then significantly repressed by ICG-induced heat and ROS, and PTX-BSA-based chemotherapy. The heat produced by photothermal therapy further accelerates the fluidity of cancer cells, thereby promoting the cancer cell uptake of photo-chemotherapy agents. In vivo data have indicated that ICG/PTX-BSA loaded CCM-LDH NSs (LIPC) significantly inhibited tumor growth under 808 nm laser. Thus, CCM modified LDH NSs would be a novel nanoplatform for CRC therapy and own the potential for further clinical translation.

## Results and discussion

### Characteristics of CCM-cloaking LDH NSs

**Fig. 1:**
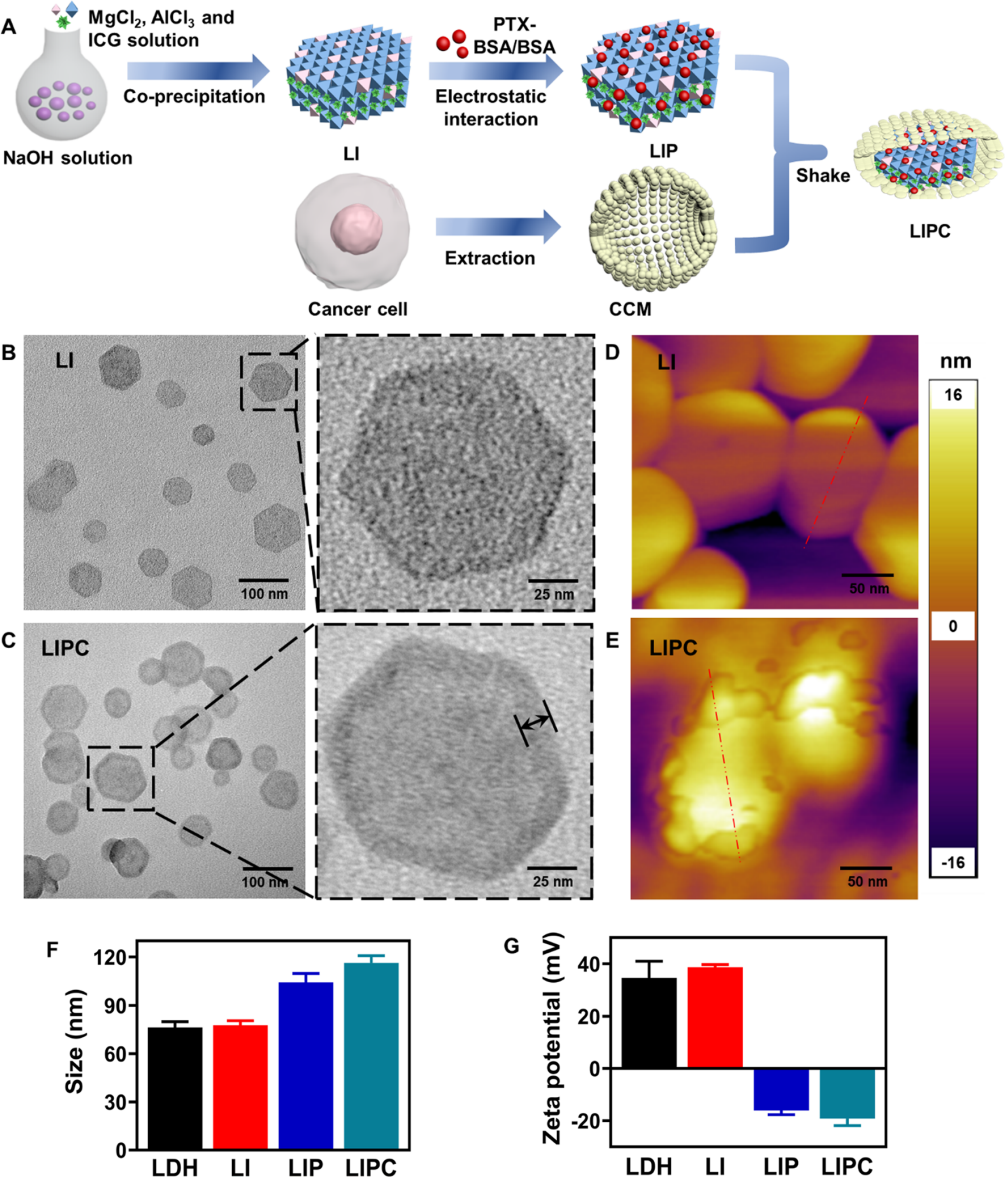
Characteristics of LIPC NSs. **A** The synthesis scheme of LIPC. **B**, **C** TEM and magnified TEM images of LI and LIPC. **D, E** AFM images of LI and LIPC. **F** Size and, **G** zeta potential of LDH, LI, LIP, and LIPC measured with DLS

In this work, we constructed CCM-LDH NSs as a novel carrier for homologous targeting photo-chemotherapy through co-loading ICG and PTX-BSA (LDH-ICG@PTX-BSA, LIP) and further coating CCM on the LIP surface (termed as LIPC, Fig. 1A). CT26 cancer cell line was used as the cancer model in this research, thus CT26 CCM was extracted. As schematically shown in Fig. 1A, LDH-ICG NSs (LI) were prepared by conventional coprecipitation as reported previously [23]. Then PTX-BSA/BSA mixture was loaded on LI to make LIP NSs via adsorption through electrostatic interactions between the positively charged LI surface and negatively charged PTX-BSA/BSA molecules. Finally, pre-extracted CCM was coated on LIP by shaking overnight to construct LIPC NSs with the mass ratio of LDH to CCM from 10:1 to 200:1. As illustrated in Additional file 1: Fig. S1, the loading efficiency of CCM on LIP reached 97.7 ± 0.5% at the mass ratio of 10:1 and further increased to almost 100% with the decreased amount of CCM (LDH:CCM = 20:1 as a typical example). The transmission electron microscopy (TEM) images (Fig. 1B) indicate that LI possessed typical hexagonal plate-like morphology with the particle lateral dimension in the range from 50 to 100 nm. After loading PTX-BSA/BSA and coating CCM, LIPC displayed a core–shell structure with the shell of 6–10 nm in length (Fig. 1C), which is in alignment with CCM-coated polymer and poly(lactic-*co*-glycolic acid) nanoparticles [14, 30]. Meanwhile, atomic force microscope (AFM) analysis revealed that the height of LIPC increased to ~ 15 nm from ~ 5 nm of LI (Additional file 1: Fig. S2). This is largely due to the encapsulation of CCM/BSA, which appears to be around 10 nm in thickness (Fig. 1D, E). The successfully developed LIPC NSs were further confirmed by the change of the average particle size and the zeta potential measured using dynamic light scattering (DLS). As shown in Fig. 1F and Table 1, the average hydrodynamic size increased from 77.6 ± 2.8 nm (LI) to 104.2 ± 5.5 nm (LIP) after PTX-BSA/BSA loading and further to 116.3 ± 4.5 nm (LIPC) due to CCM coating. Meanwhile, the zeta potential verified that positively charged LI (38.7 ± 1.0 mV) were turned into negatively charged LIP (− 16.1 ± 1.6 mV) after electrostatically absorbing PTX-BSA/BSA and then more negatively charged LIPC (− 19.2 ± 2.7 mV) after further incorporating CCM (Fig. 1G). Note that negatively charged surface could reduce the aggaragation of nanoparticles in cell medium and blood, which could finally enhance tumor targeting and treatment efficiency. The polydispersity index (PDI) indicated excellent dispersion and narrow particle size distribution of these LDH NSs (Table 1). Collectively, CT26-derived CCM was coated on the LIP surface successfully.

**Table 1:** Size, PDI, zeta potential, loading efficiency, and capacity of LDH-based NSs

Sample	Size (d. nm)	PDI	Zeta potential (mV)	Loading efficiency of ICG/PTX-BSA (%)^c^	Loading capacity of ICG/PTX-BSA (%)^c^
LDH	76.3 ± 3.6	0.324	34.6 ± 6.4	−/−	−/−
LI	77.6 ± 2.8	0.246	38.7 ± 1.0	61.8 ± 1.2/–	4.7 ± 0.1/−
LIP^a^	104.2 ± 5.5	0.265	− 16.1 ± 1.6	61.7 ± 0.2/99.9 ± 0.1	4.6 ± 0.1/2.3 ± 0.1
LIPC^a,b^	116.3 ± 4.5	0.217	− 19.2 ± 2.7	61.7 ± 0.1/99.9 ± 0.1	4.6 ± 0.1/2.3 ± 0.1

^a^BSA was employed for coating LIP and LIPC but was not calculated in loading capacity

^b^The loading efficiency and capacity of CCM on LIPC were 98.6% and 4.7%, respectively at the mass ratio of LDH to CCM of 20:1

^c^Loading efficiency (wt%) = Mass of ICG/PTX-BSA in NSs/Mass of ICG/PTX-BSA added × 100%; Loading capacity (wt%) = Mass of ICG/PTX-BSA in NSs/Mass of NSs × 100%

The loading efficiency and capacity of ICG were determined based on the standard curve of ICG (Additional file 1: Fig. S3) and that of PTX-BSA was estimated by BCA protein assay. As listed in Table 1, the loading efficiency and capacity of ICG on LIPC were 61.7% and 4.6%, respectively. The loading efficiency of PTX-BSA on LIPC reached almost 100% with the loading capacity of 2.3%. Both loaded therapeutics were adequate for CRC treatment [26]. Note that the loading of ICG and PTX-BSA on LIPC could be adjustable more or less accordingly. The colloidal stability of LIPC in PBS was verified by monitoring the size distribution using DLS. The size of LIPC in PBS remained unchanged within 24 h. By contrast, the size of LI increased from 77.6 ± 2.8 nm at 0 h to 531.2 ± 98.7 nm at 6 h, and further to a few micrometers at 12 and 24 h, manifesting severe aggregation of LI in PBS (Additional file 1: Fig. S4). Moreover, the size distribution of LIPC exhibited negligible change in PBS within 14 days (Additional file 1: Fig. S5), indicating LIPC has excellent colloid stability due to BSA and CCM stabilization [31].

Subsequently, we investigated the CCM loading efficiency and capacity at the LDH to CCM mass ratio from 10:1 to 200:1, using BCA protein assay kit. The loading efficiency of CCM on LIPC reached approximately 100% at the mass ratio above 20:1 (Additional file 1: Fig. S1). Furthermore, the size and the zeta potential of LIPC showed negligible variation at the LDH:CCM mass ratios used in this research (Additional file 1: Table. S1), suggesting LIP can load a certain amount of CCM on the surface with the loading efficiency of nearly 100%.

### Photothermal performance and payload release of LIPC NSs

**Fig. 2:**
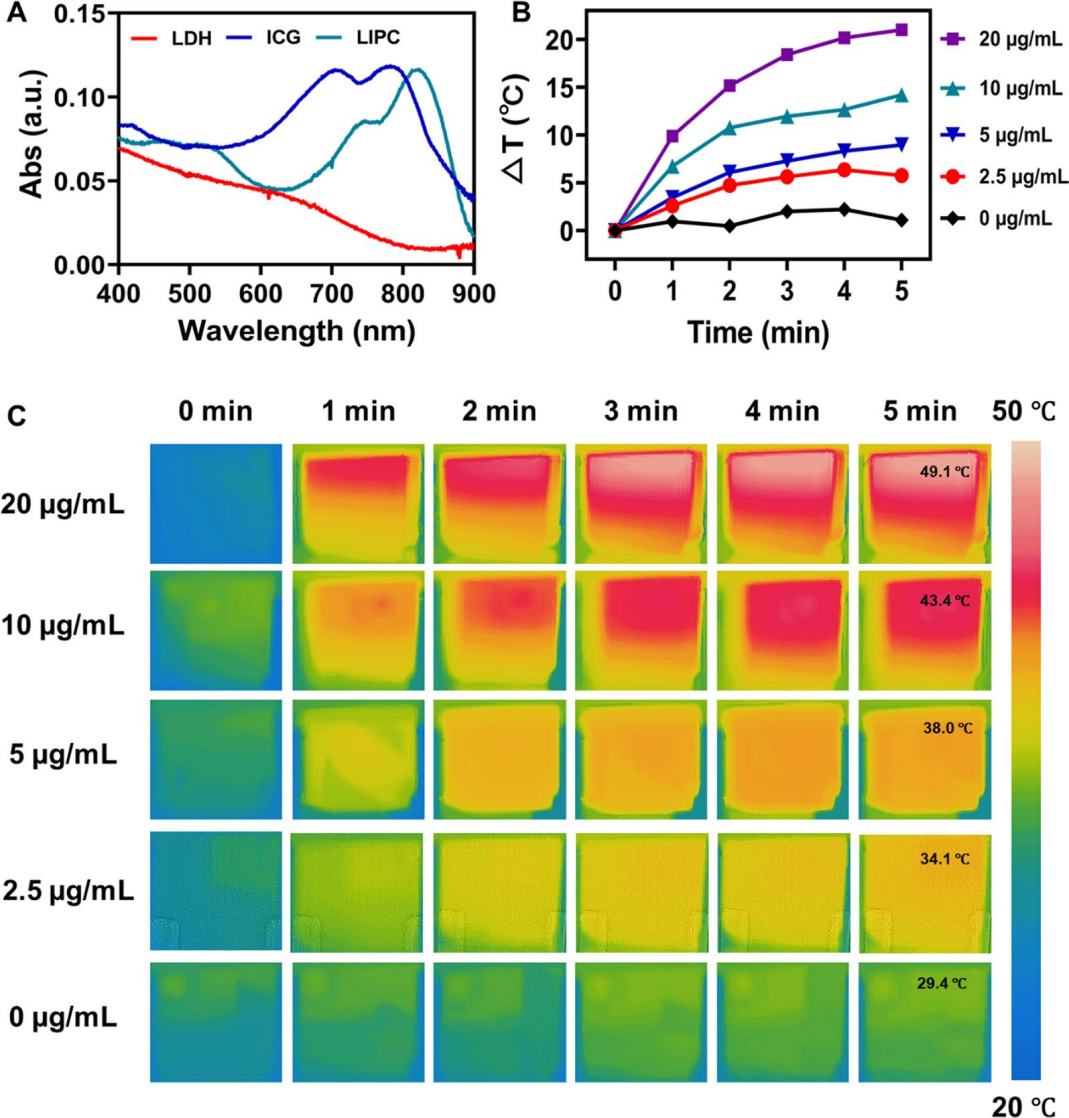
Photothermal performance of LIPC upon laser irradiation. **A** UV–vis absorbance spectra of LIPC, LDH and free ICG. **B** Temperature change profiles and **C** photothermal images of LIPC under laser irradiation (808 nm, 0.5 W/cm^2^) for 5 min. The concentrations of ICG were 0–20 µg/mL and the mass ratio of LDH to CCM was 20:1

The photothermal property of LIPC (LDH:CCM = 20:1) was examined by measuring the temperature of the LIPC suspension heated upon 808 nm laser irradiation. Interestingly, one major UV–vis absorption peak of LIPC was shifted to 820 nm from 780 nm of free ICG (Fig. 2A). This shift may make LIPC more effective for photothermal conversion when 808 nm NIR laser is used. Subsequently, photothermal imaging and the temperature change of LIPC suspension under laser irradiation (0.5 W/cm^2^) (laser was denoted as ‘L’ subsequently) for 5 min was recorded. As shown in Fig. 2B, C, the temperature was increased in a time- and concentration-dependent manner. In particular, the temperature of LIPC suspension was elevated by 21.3 °C under laser irradiation for 5 min at the ICG concentration of 20 µg/mL. Consistently, the temperature change of LIP suspension showed a similar profile, revealing the camouflage of CCM did not influence the photothermal performance of the LDH-ICG NSs, as reported elsewhere (Additional file 1: Fig. S6) [31]. In comparison, the temperature of free ICG solution was increased slowly upon the similar irradiation, manifesting the photothermal property of LIPC was improved once ICG was intercalated into the LDH interlayer (Additional file 1: Figs. S7, S8). Furthermore, the photothermal stability of LIPC was demonstrated by the negligible peak temperature variation, while the peak temperature of free ICG solution was significantly reduced during four ‘on–off’ cycles of laser irradiation (Additional file 1: Fig. S9), due to the protection of photodegradable ICG by the LDH framework. These data demonstrate that LIPC NSs have excellent photothermal performance with improved photostability.

The drug release (represented by ICG) percentage of LIPC under laser irradiation in acidic (pH = 6.0 simulating tumor microenvironment: TME) and neutral (pH = 7.4 simulating physiological condition) environments was further investigated. As expected, only 24.3% of ICG was released at pH = 7.4 within 120 min but the percentage increased to 76.6% at pH = 6.0, manifesting TME may help release the drug (ICG) via dissolution of LIPC (Additional file 1: Fig. S10). Moreover, similar laser irradiation improved the ICG release by ~ 20% both in acidic and neutral buffers within 120 min.

### Hyperthermia-facilitated homologous targeting and immune escaping ability of LIPC

**Fig. 3:**
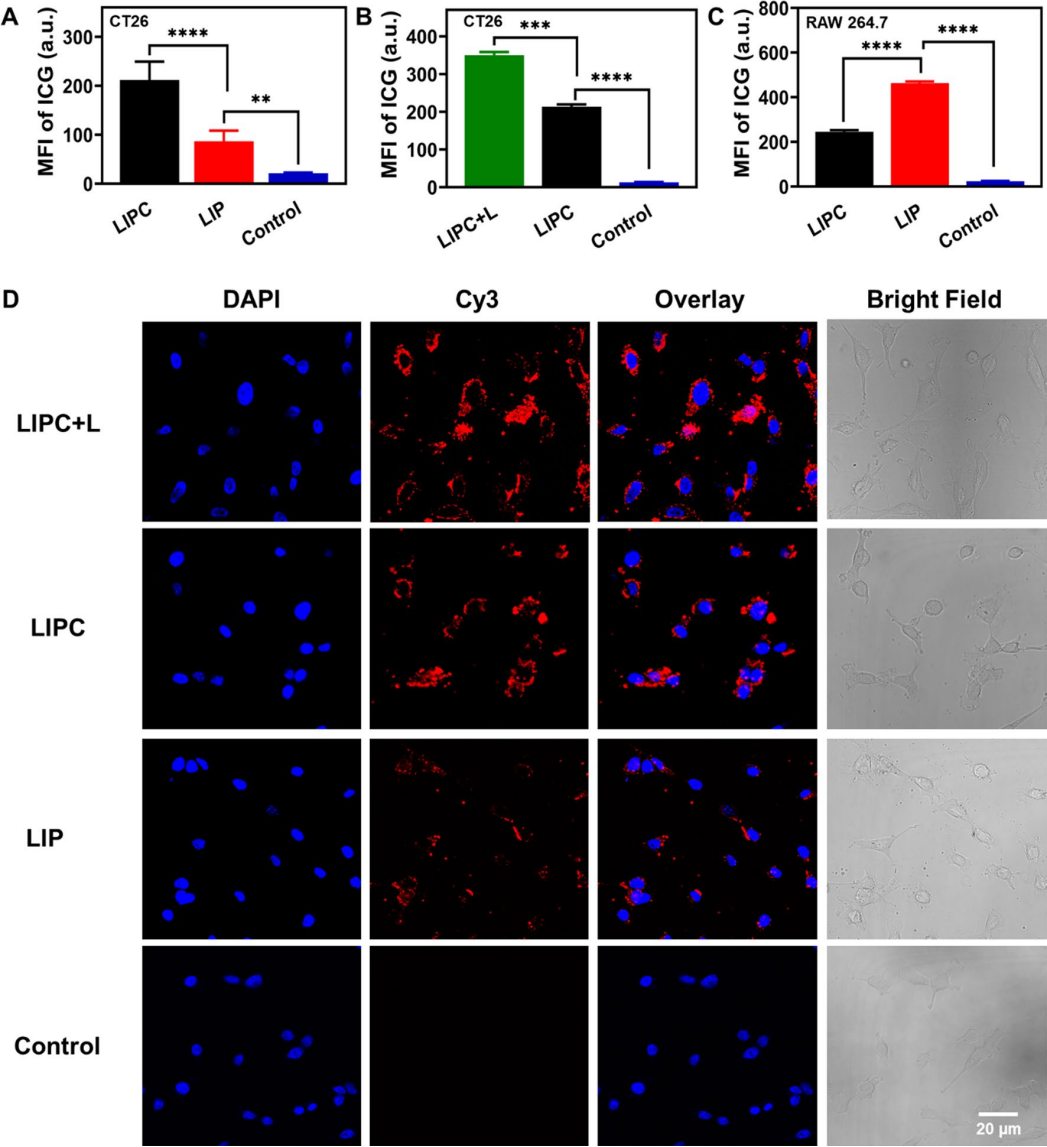
Hyperthermia-assisted homologous targeting and immune escaping ability of LIPC. **A** The MFI of CT26 cells treated with LIPC and LIP for 4 h measured by flow cytometry. **B** The MFI of CT26 cells treated with LIPC for 4 h, laser irradiation (808 nm, 0.5 W/cm^2^) for 5 min and incubated for another 1 h. **C** The MFI of RAW 264.7 cells treated with LIPC and LIP for 4 h. The concentration of ICG was 0.5 µg/mL. **D** Cell internalization of Cy3-dsDNA tagged LIPC and LIP after incubation for 4 h, laser irradiation (808 nm, 0.5 W/cm^2^) for 5 min and incubation for another 1 h. The concentration of Cy3-dsDNA was 40 nM. The mass ratio of LDH to CCM was 20:1. Scale bar: 20 µm. n = 3

Hyperthermia-boosted homologous targeting efficiency of CCM was evaluated using cellular internalization assay. As illustrated in Fig. 3A (LDH:CCM = 20:1 as a typical example), the mean fluorescence intensity (MFI) of LIPC-incubated CT26 cells was significantly enhanced by 2.4 times that of corresponding LDH NSs without CCM encapsulation, manifesting that even a small amount CCM on the LIPC surface improved the homologous targeting and cellular uptake (Additional file 1: Fig. S11A). The targeting was also dependent on the amount of CCM loaded on the surface of LDH NSs. As shown in Additional file 1: Fig. S11A, the cellular uptake of LIPC (i.e. the MFI of ICG) was increased when the LDH to CCM mass ratio changed from 200:1 to 20:1. Note that the MFI value was very similar for LIPC at 20:1 and 10:1. Consistently, the positive cell percentage reached 93.1% and 90.4% in LIPC-treated cells at the mass ratio of 10:1 and 20:1, approximately 2.8–2.9 times that of LIP-treated cells (32.1%) (Additional file 1: Fig. S11B). The targeting capacity of LIPC at 20:1 is overall very similar to that reported by Xiao et al. [32]. Thus, the optimal LDH:CCM mass ratio for homologous targeting seemed to be 20:1, which was used as the typical LIPC in the following experiments.

The targeting delivery efficacy of LIPC was further enhanced by laser irradiation-induced heat. As exhibited in Fig. 3B, the MFI of LIPC-treated cells (LIPC + L) was elevated significantly after laser irradiation compared with the corresponding group (LIPC), demonstrating photothermal heating promoted the targeting delivery of LIPC toward CT26 cells, which is expected to improve the therapeutic efficacy of CRC. The percentage of positive cells showed a similar trend (Additional file 1: Fig. S12). In summary, laser-initiated hyperthermia accelerated the fluxion of the cell membrane and loaded-CCM, resulting in rapid uptake of LIPC NSs by CT26 cells.

Another notable property is that CCM provided LDH NSs with the immune escaping ability [33], as validated by the uptake of LIPC and LIP using RAW 264.7 cells. As shown in Fig. 3C and Additional file 1: Fig. S13, the internalized ICG amount and the ICG-positive cell percentage were reduced considerably in the LIPC group (245.5 ± 7.8; 53.1%) in comparison with the LIP group (463.9 ± 7.2; 85.9%). Subsequently, the specificity of CCM targeting CT26 cells was further proved by the reduced uptake by B16F0 and HEK-293T cells. As exhibited in Additional file 1: Figs. S14, S15, the MFI and the positive percentage of B16F0/HEK-293T cells in the LIPC group (79.6 ± 6.8; 8.9%/98.6 ± 1.4; 3.3%) were significantly decreased compared with the LIP group (141.4 ± 9.2; 38.7%/146.0 ± 2.1; 26.0%). This observation demonstrates that CT26 CCM inhibits the uptake of LDH NSs by health and other cancer cells, which mitigates the off-target delivery of therapeutic LDH NSs and subsequent side effects, which is similar to that reported elsewhere for CCM encapsulated nanoparticles [32].

Heat facilitated targeting delviery was further confirmed using Cy3-dsDNA-tagged LIPC NSs by flow cytometry and laser scanning confocal microscope (LSCM). As previously reported, Cy3-dsDNA was used specifically for this imaging to show LDH NS internalization and full loading at the LDH:Cy3-dsDNA mass ratio of 3:1 [28]. In consistency with ICG uptake, the MFI of Cy3 and the percentage of Cy3-positive CT26 cells incubated with LIPC were enhanced strikingly in comparison with that using LIP and further boosted by laser irradiation (Additional file 1: Fig. S16). Meanwhile, the red fluorescence signal (Cy3) of LIPC + L treated CT26 cells were significantly stronger than that of LIPC and LIP treated groups (Fig. 3D). Collectively, LIPC exhibited very high homologous targeting efficiency toward CRC cells, but efficiently escaped from the macrophage clearance and significantly reduced the uptake by normal health and other cancer cells (Additional file 1: Figs. S14, S15).

### Enhanced cytotoxicity of LIPC upon 808 nm laser irradiation

**Fig. 4:**
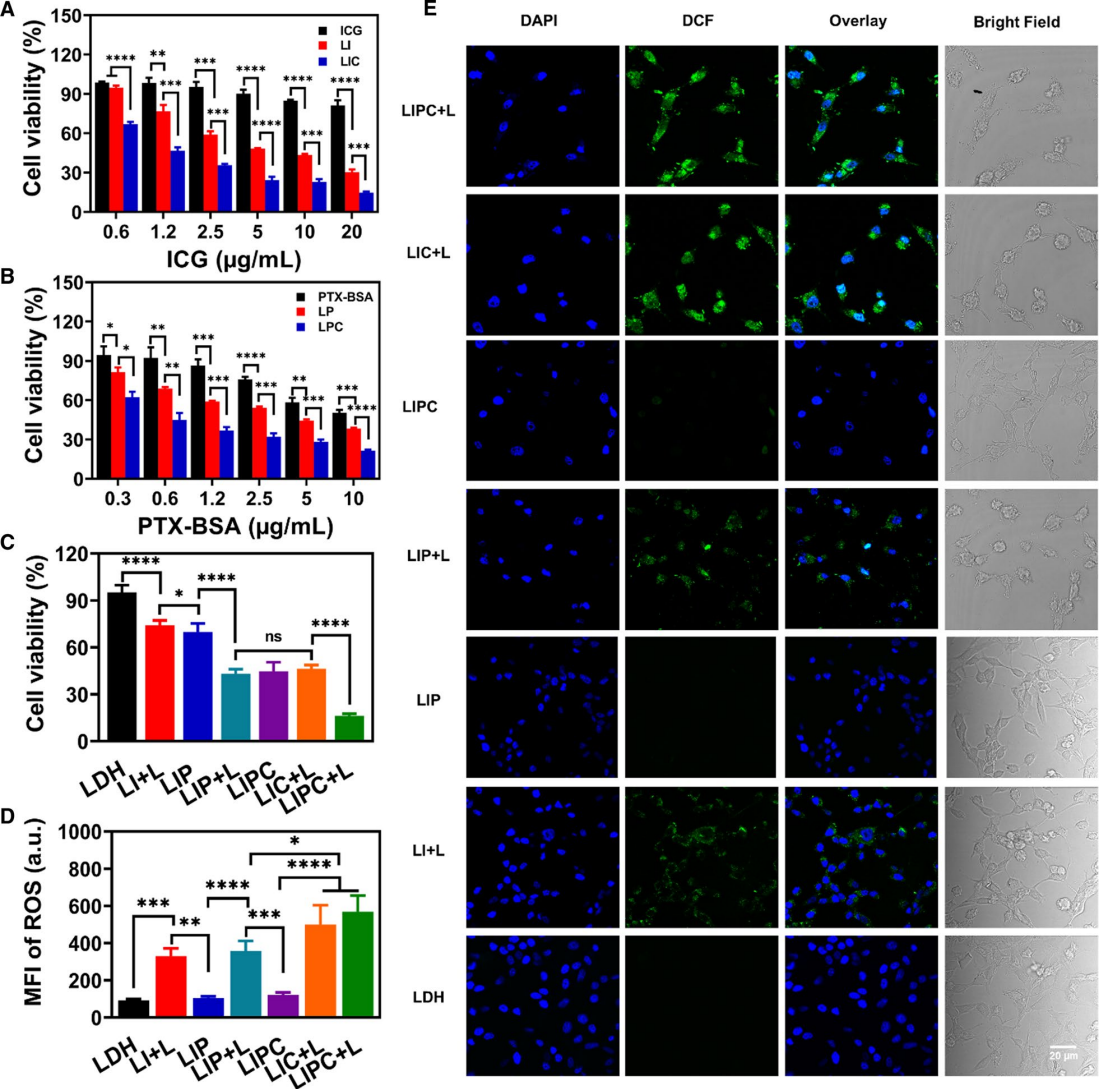
Cytotoxicity of LIPC in CT26 cells under 808 nm laser. **A** Cell viability of CT26 cells treated with LIC, LI, and free ICG for 18 h, 808 nm laser irradiation (0.5 W/cm^2^, 5 min) and cultrured for another 6 h, and **B** with LPC, LP, and free PTX-BSA for 24 h. **C** Cell viability of CT26 cells with various treatments. The concentration of ICG, PTX-BSA, and CCM were 1.2, 0.6, and 2.5 µg/mL, respectively. **D, E** ROS generation in CT26 cells with various treatments. Scale bar: 20 µm

In vitro anti-tumor efficiency of LIPC was determined by MTT assay. As exhibited in Fig. 4A, cytotoxicity of LI and LIC was dose-dependent under 808 nm laser (0.5 W/cm^2^, 5 min) and much higher than that of free ICG, with the IC_50_ value being 5.78 and 1.22 µg/mL (vs. 143.5 µg/mL for free ICG), respectively, indicating that (1) LDH NSs as a carrier remarkably facilitate cellular uptake [34], and (2) CCM significantly improves the cellular uptake by homologous targeting. As reported by Chen et al., ICG loaded with molybdenum selenide nanoparticles showed the IC_50_ value of approximately 12.5 µg/mL, which is considerably higher than that of LI and LIC, further reflecting the facilitation of LDH for cellular uptake and targeting delivery efficiency of CCM [34, 35]. Note that LIC, LI, and ICG had nearly no influence on cell proliferation in the dark (Additional file 1: Fig. S17).

Coincidentally, CCM improved the therapeutic efficiency of PTX-BSA loaded LDH (LPC), with IC_50_ decreased from 9.04 µg/mL for free PTX-BSA to 3.35 µg/mL for LP and then to 0.57 µg/mL for LPC (Fig. 4B), which are significantly lower than that of dendrimer and lipid loaded PTX nanoparticles [36, 37]. It is reported that PTX-BSA forms a 130-nm nanoparticle. However, PTX-BSA nanoparticles quickly disassembly into PTX-BSA molecules (⁓10 nm) at the concentration below 100 µg/mL [26]. These PTX-BSA molecules are supposed to adsorb onto the surface of LDH NSs, and their cellular uptake is facilitated by LDH NSs and further enhanced by CCM targeting, similarly to the ICG uptake by CT26 cells.

Based on the IC_50_ values, LIPC with the ICG:PTX-BSA mass ratio of nearly 1.22:0.57 (i.e. 2:1) was determined, which would be an optimal one and was selected for the following experiments. As exhibited in Fig. 4C, cytotoxicity was enhanced tremendously in LIPC with laser (LIPC + L) compared with LIC with laser (LIC + L) and LIPC (without laser). The combination index (CI) was calculated (Additional file 1: Table. S2) at the same equivalent concentrations of ICG and PTX-BSA. Significantly, the CI of combined photo-chemotherapy in LIP + L and LIPC + L groups were 1.20 and 1.27, respectively, revealing mild synergy between ICG and PTX-BSA for inhibiting cancer cell proliferation, which is primarily attributed to LDH-enhanced and CCM-boosted delivery of photo-chemotherapeutic agents to cancer cells.

The production of ROS due to the photodynamic effect of ICG in LIPC-treated cells upon laser irradiation was further detected by DCFH-DA assay. Flow cytometry data (Fig. 4D) revealed that LIPC, LIC, LIP and LI induced the generation of much more ROS under laser irradiation (‘ + L’). More importantly, the homologous targeting of CCM further boosted the ROS production, similar to the previous report [38]. The percentage of DCF-positive cells exhibited the same trend (Additional file 1: Fig. S18). Moreover, the fluorescence signal of DCF in LIPC + L treated cells was much stronger than that of LIP + L and LI + L group (Fig. 4E), further demonstrating the high targeting capacity of CCM. Note that there was negligible fluorescence signal in cells treated with LIPC without laser irradiation, disclosing that ROS overgeneration was initiated mainly by laser irradiation (Fig. 4E). Taken together, the enhanced phototherapy efficiency of LIPC is largely attributed to LDH facilitation to cellular uptake and CCM targeting delivery, which both promoted the PTT/PDT and chemotherapy to efficiently induce cancer cell apoptosis.

### In vivo homologous targeting tumor accumulation of LIPC

**Fig. 5:**
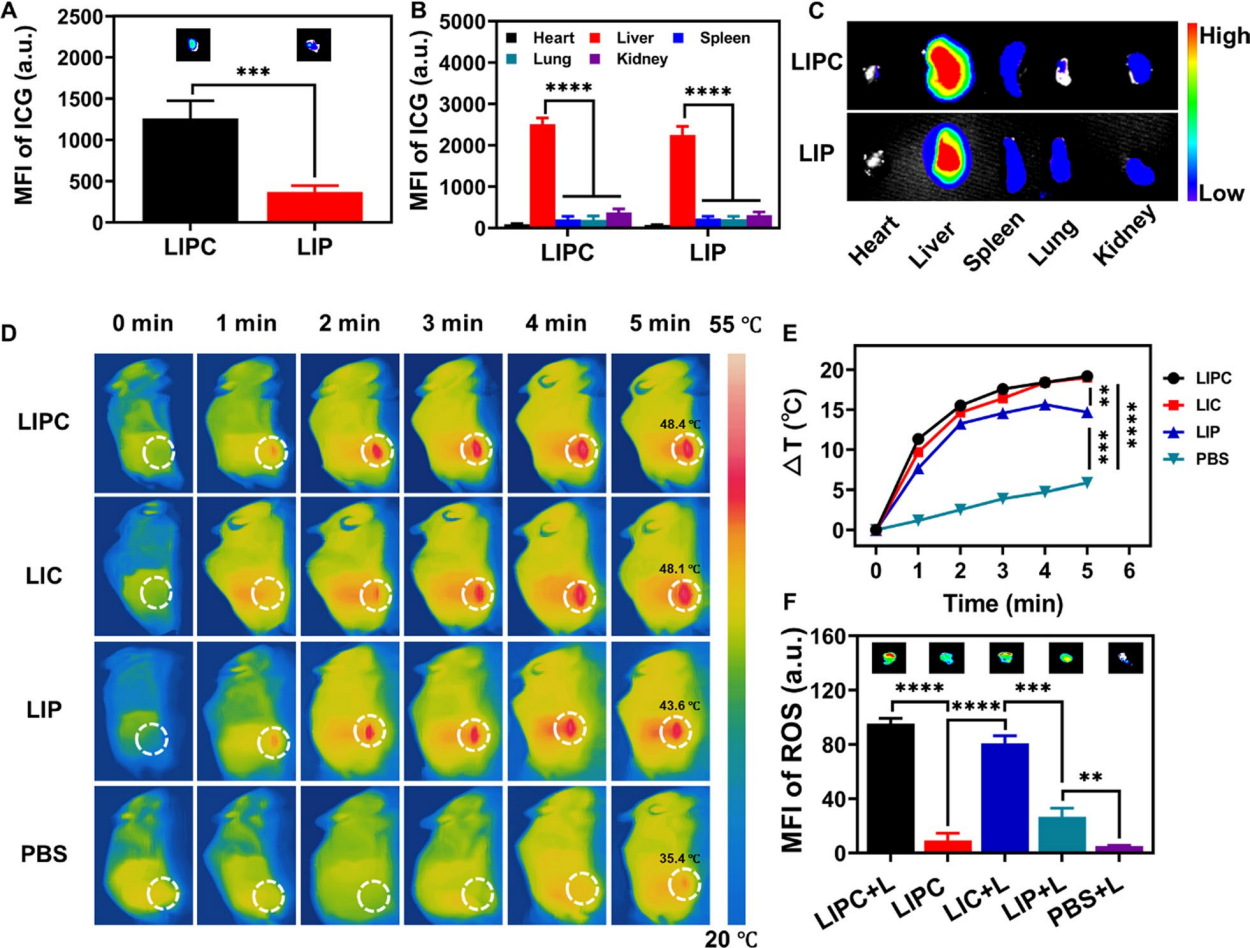
Biodistribution, photothermal performance and ROS production in CT26 tumor bearing mice. **A** The MFI of ICG in the tumor sites at 24 h post-injection. The inner photos were fluorescence images of tumors. **B** The MFI of ICG in the major tissues. **C** Ex vivo fluorescence images of the tissues collected at 24 h post-injection. **D** Photothermal images of the mice treated with LIPC, LIC, LIP, and PBS under laser irradiation (808 nm, 0.5 W/cm^2^) for 5 min. The laser irradiation was performed at 24 h after intravenous injection. **E** Temperature change profiles of the mice. **F** The generation of ROS in mice upon different treatments. The inserted photos are the fluorescence images of ROS in the tumor tissues

To investigate the homologous targeting and photo-chemotherapy of LIPC in vivo, a colon tumor model was established by subcutaneous injection of CT26 cells (2 × 10^6^ cells, 100 µL) on the right flank of the mice. When the tumors grew to appropriately 100 mm^3^, CT26 tumor-bearing mice were injected with LIPC and LIP (1.2 mg/kg of ICG, 0.6 mg/kg PTX-BSA, and 1.2 mg/kg of CCM) intravenously to examine the particle biodistribution. At 24 h post-injection, the mice were sacrificed, the tumors collected for homologous targeting evaluation, and the organs harvested for biodistribution analysis using ex vivo ICG fluorescence imaging. Comparing the MFI of ICG in the tumor, we found that CCM significantly increased the tumor accumulation of LIPC, as evidenced by more than 3 times fluorescence signals in the tumor tissues compared to that of LIP (Fig. 5A). Owning to CCM homotypic targeting, LIPC achieved greater accumulation at tumor sites with a lower concentration of ICG (1.2 mg/kg of ICG) in comparison with HAS carrying ICG (2 mg/kg of ICG) [39]. Previous studies have demonstrated that the accumulation of LDH nanoparticles coated with BSA in the tumor tissues is appropriately 3.0% of the injection dose at 24 h post-injection [40]. Thus, the LIPC accumulation in the tumor would be up to 9.0% of the injected dose, much higher than that of other nanoparticles (about 1.0%). The MFI of ICG in the organs (Fig. 5B) and the fluorescence images (Fig. 5C) demonstrate that LIPC and LIP were mainly captured in the liver, similar to the previous report for LDH NSs [22].

Subsequently, photothermal heating of LIPC, LIC, and LIP upon 808 nm laser irradiation at 0.5 W/cm^2^ for 5 min was evaluated. As reported previously, LDH-based nanoparticles reach the highest accumulation in the tumor tissue at 24 h post intravenous injection [22, 24]. Thus, 24 h post injection was selected as the time point to conduct the phototherapy. As shown in Fig. 5D, E, the temperature was increased to 48.4 °C and 48.1 °C for LIPC and LIC at 24 h post-injection, respectively, which is significantly higher than 43.6 °C for LIP. This temperature increase is even comparable to that using much higher laser power (2 W/cm^2^) for 5 min for HAS NPs at a high dose of ICG (2.0 mg/kg), as reported [39]. Note that heat dissipation from the hotter region upon PTT is more rapid than that from the cooler region, so the difference should be even more significant if there were no heat dissipation. To demonstrate the safety of laser to the healthy tissue around the tumor, we tracked the temperature of LIPC + L group at 5 min, as shown in Additional file 1: Fig. S19A. Both the temperature (33.4 °C/34.5 °C) at points 1/5 (healthy tissues) and 2/4 (43.7 °C/43.5 °C, region between the tumor and the healthy tissue) should be well tolerated by adjacent healthy tissues in comparison with that at 3 (48.9 °C, tumor center). As further illustrated in Fig. 5F, the ROS generation of LIPC + L (95 units) and LIC + L (81 units) group in the tumor tissues was much more than that generated by LIP + L (27 units), which should proportionally represent the amount of LDH NSs accumulated in the tumor tissues. Moreover, the production of heat and ROS is further boosted primarily by the accumulation of more LIPC NSs in the tumor tissue due to homotypic tumor self-recognition, evasion from immune clearance, and prolonged blood circulation [41, 42]. Altogether, as a delivery carrier, LDH-CCM benefits high accumulation of anti-tumor agents and may improve the anti-tumor treatment efficiency [43].

To fulfill the optimal treatment effect and minimal hyperthermic damage to the adjacent healthy tissues, the laser power density was optimized in the mouse model. As exhibited in Additional file 1: Fig. S20A, the temperature of tumor tissues was increased by 28.4 °C, 19.8 °C, and 11.7 °C upon 808 nm irradiation at 0.7, 0.5 and 0.3 W/cm^2^ for 5 min at 24 h post-injection of LIPC, respectively. Since the temperature (54.9 °C) at the power of 0.7 W/cm^2^ would damage the surrounding skin while that (42.3 °C) at 0.3 W/cm^2^ would be insufficient to retard tumor progression, the power density at 0.5 W/cm^2^ appeared to heat to a suitable temperature (48.4 °C) (Additional file 1: Fig. S20B) and seemed the optimal laser power.

### In vivo homologous targeting enhanced anti-tumor therapy

**Fig. 6:**
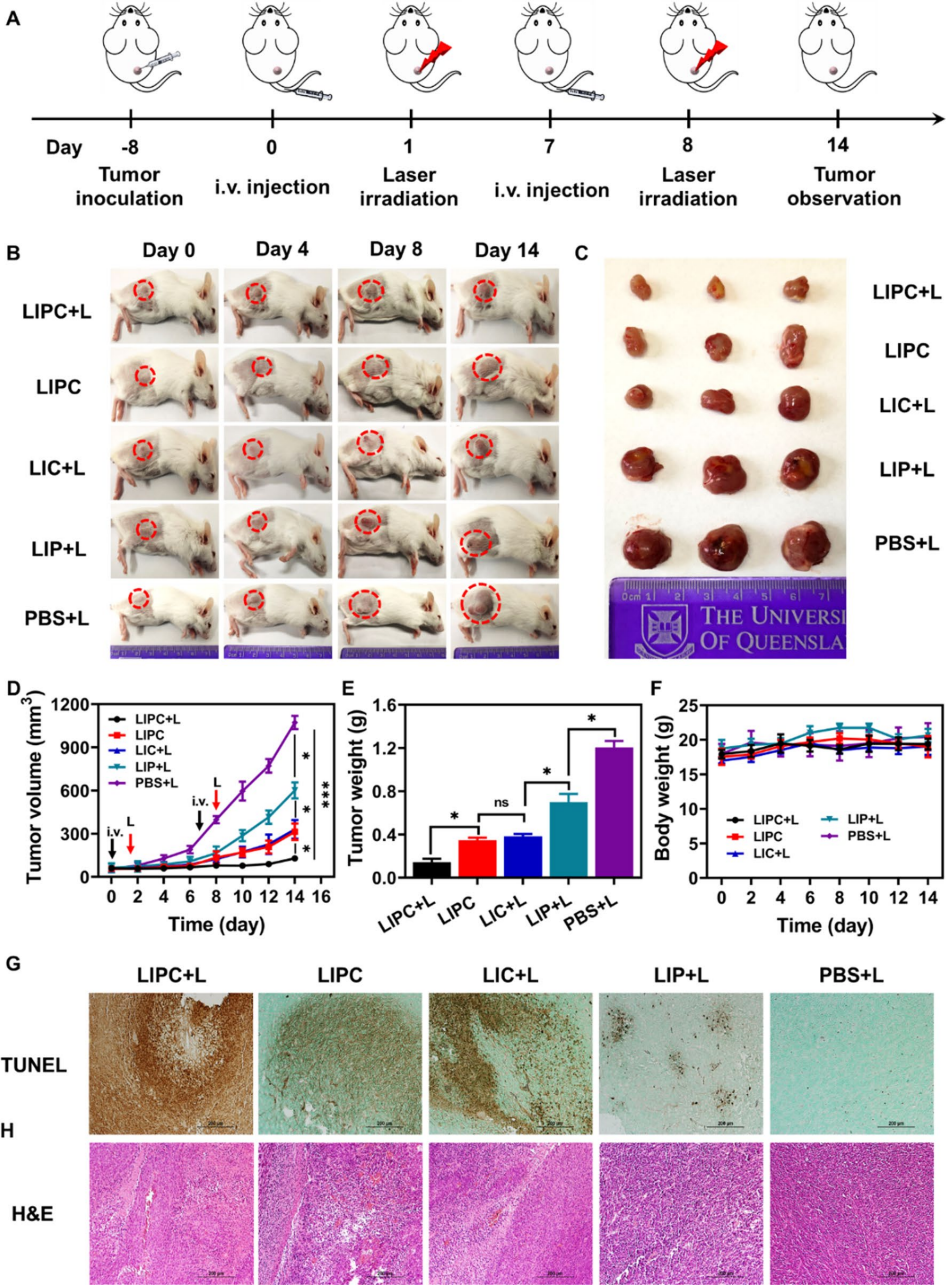
Therapeutic effects of LIPC in vivo. **A** Schematic illustration of the experimental procedure. **B** The images of mice at day 0, 4, 8, and 14 upon different treatments. **C** The images of typical tumors collected at day 14. **D** Tumor volume change profile of the mice. **E** The average tumor weight of the mice at day 14. **F** Body weight of the mice. **G** TUNEL and **H** H&E staining of the tumors collected at day 14

Inspired by the outstanding homologous targeting and photo-chemotherapy properties of LIPC, in vivo therapeutic efficacy was further examined in CT26 tumor-bearing mice. When the tumor volume reached 50–100 mm^3^, the mice were randomly divided into 5 groups (n = 5) receiving following treatments, i.e. (1) LIPC + L, (2) LIPC, (3) LIP + L, (4) LIC + L, and (5) PBS + L. As illustrated in Fig. 6A, LIPC, LIC, LIP (1.2 mg/kg of ICG, 0.6 mg/kg of PTX-BSA, and 1.2 mg/kg of CCM) and PBS were injected intravenously twice at day 0 and 7 after the establishment of CT26 tumor models, respectively. At 24 h post-injection, the mouse tumors were irradiated with 808 nm laser (0.5 W/cm^2^) for 5 min (i.e. at day 1 and 8). The tumor volume and body weight were monitored every other day for 14 days. On day 14, the mice were sacrificed, and tumors and organs were harvested for histopathological analysis.

As shown in Fig. 6B, C, the tumor grew rapidly in the PBS + L treated mice, similar to the previous reported [44], revealing that low power light irradiation did not influence tumor progression. The tumor inhibition in the LIP + L group was limited, with the tumor growth inhibition (TGI) of 44% at day 14 (Fig. 6D, E). In comparison, tumor progression was attenuated intensively in both LIC + L (TGI = 69.2%) and LIPC (TGI = 70.7%) groups, demonstrating that both singular phototherapy (ICG) and chemotherapy (BSA-PTX in LIPC, supposing that ICG did not contribute without laser) significantly suppressed tumor growth via CCM-targeting delivery to the tumor tissues. This inhibition efficacy is much better than that reported for ICG conjugated onto gold nanospheres which achieved similar TGI at a much higher dose (5 mg/kg of ICG) on colorectal cancer [45]. Similarly, the TGI in liposome loating PTX was also similar in CT26 tumor-bearing mice when intravenously injected with liposome-PTX at 8 mg/kg of PTX, synchronously indicating improved therapeutic efficiency due to BSA-PTX loading and CCM coating in LIPC NSs [44].

Very remarkably, tumor growth was alleviated in the LIPC + L group of mice, with the TGI up to 88% at day 14. Notably, the TGI of LIPC + L group (88%) was 2 times higher than that of the corresponding LIP + L group (44%), indicating that the therapeutic efficacy of anti-tumor agents (ICG for phototherapy whereas Abraxane for chemotherapy) was improved by increased tumor targeting due to CCM encapsulation. In addition, the inhibition is consistent with the weight of tumors collected at day 14 (Fig. 6E). The terminal deoxynucleotidyl transferase-mediated dUTP nick-end labeling (TUNEL) staining of the tumors revealed that there was much more enhanced tissue lesion in LIPC + L group of mice compared to the rest groups (Fig. 6G). Specifically, cell apoptosis of this group of tumor tissues was boosted dramatically with CCM coating and laser irradiation compared to that of LIPC and LIC + L groups. These data collectively suggest the synergistic effect of photo-chemotherapy via targeted accumulation in the tumor tissues and minimized clearance by macrophage during systemic circulation.

It is worth noting that all treatments had no impact on the bodyweight of the mice (Fig. 6F), and showed no toxicity towards the major organs (including heart, liver, spleen, lung, and kidney) (Fig. 6H and Additional file 1: Fig. S21), suggesting CCM-LDH NSs have good biocompatibility. In a word, LIPC NSs have exhibited potential anti-tumor effects in vivo without systemic toxicity.

## Conclusion

In summary, hyperthermia-assisted homologously targeting photo-chemotherapy was achieved by CCM-camouflaged ICG/PTX-BSA co-loaded LDH NSs (LIPC). Due to the natural characteristics of CCM and LDH NSs, LIPC showed the most efficient and targeting delivery to homologous tumor cells and tumor tissues. Interestingly, photothermal heat boosted the cellular uptake of photo-chemotherapy agents by cancer cells, enhancing photo-chemotherapeutic cytotoxicity in a synergetic way. Heat (PTT) and ROS (PDT) generated under NIR laser irradiation together with released drug PTX-BSA efficiently induced cancer cell apoptosis. In vivo experiments validated improved inhibition of tumor growth at lower doses of therapeutics under mild laser radiation. Hence, CCM-cloaked LDH NSs can serve as a potential platform for combination cancer therapy.

## Materials and methods

### Materials

MgCl_2_·6H_2_O, AlCl_3_·6H_2_O, and NaOH were obtained from Chem-Supply. Indocyanine green (ICG), paclitaxel-bovine serum albumin (PTX-BSA), ethylenediaminetetraacetic acid (EDTA), and dichlorofluorescein diacetate (DCFH-DA) were purchased from Sigma-Aldrich. Bovine serum albumin (BSA), Dulbecco’s Modified Eagle Medium (DMEM), 3-(4,5-dimethylthiazol-2-yl)-2,5-diphenyltetrazolium bromide (MTT), penicillin/streptomycin and 4′,6-diamidino-2-phenylindole (DAPI) dye were purchased from Thermal Fisher. Phosphate buffered saline and fetal bovine serum (FBS) were acquired from BioWhitter. Deionized Milli-Q water was used in all experiments.

### Extraction of CCM

CT26 cells were grown with DMEM medium supplemented with 10% FBS, 100 U/mL penicillin, and 100 μg/mL streptomycin in a humidified atmosphere of 5% CO_2_ at 37 °C. When the confluence reached 80%, the cells were detached with 2 mM EDTA containing PBS. Next, the cells were collected, washed with PBS for 3 times, and re-suspended in a hypotonic lysing buffer consisting of 20 mM Tris–HCl, 10 mM KCl, 2 mM MgCl_2,_ and 1 EDTA-free mini protease inhibitor tablet. Subsequently, repeated freeze–thaw with sonication for 10 times was applied to lyse the cells. Ultimately, the cell debris was centrifuged at 15,000 g for 30 min and CCM in the supernatant was collected for further use. The concentration of CCM was determined by BCA protein assay kit.

### Preparation of ICG-loaded LDH NSs (LI)

A solution containing MgCl_2_ (0.6 M), AlCl_3_ (0.2 M) and ICG (4 mM) in 5 mL of deionized water was quickly added into NaOH solution (20 mL, 0.4 M) under vigorous stirring. After stirring vigorously for 20 min, LI was collected via centrifugation and washed twice with 20 ml of deionized water. Then LI slurry was resuspended in deionized water (20 mL) and stood by at room temperature for 3 days to prepare monodispersed LI suspension.

### Synthesis of PTX-BSA-loaded LI (LIP)

An equal volume of PTX-BSA solution (24 µg/mL, 10 wt % PTX in PTX-BSA) was slowly added to LI suspension (1 mg/mL) under vigorous stirring and the stirring was continued for 30 min based on our previous report [26]. Subsequently, the mixture was added to the equal volume of BSA (5 mg/mL) to obtain LIP. After centrifuging at 15,000 rpm for 30 min, LIP pellet was collected and re-suspended in deionized water.

### Preparation of CCM-coated LIP (LIPC)

A certain volume of CCM suspension was slowly added to LIP suspension under vigorous stirring and then shaken at 4 ℃ overnight. To optimize the amount of CCM to prepare LIPC, different mass ratios of LDH to CCM were investigated. Briefly, 100, 50, 20, 10 and 5 µL CCM (1 mg/mL) were added to 1 mL of LIP mixture (LDH: CCM = 10:1, 20:1, 50:1, 100:1 and 200:1). The loading efficiency and the amount of CCM on LIP were calculated based on the analysis of free CCM in the suspension by the BCA protein assay kit.

### Photothermal performance

Solutions with LIPC, LI, and free ICG at the ICG concentration of 20, 10, 5, 2.5, and 0 µg/mL were irradiated with laser (808 nm, 0.5 W/cm^2^) for 5 min. Photothermal images (PTI) were recorded by an infrared camera and the temperature was transformed accordingly.

### ICG release from LIPC

LIPC suspension (ICG = 500 µg, 3 mL) was added into the dialysis bag and immersed in 50 mL PBS (pH = 7.4) and citric acid buffer (pH = 6.0). Buffer (200 µL) was withdrawn at 20, 40, 60, 80, 100, 120, and 240 min for measuring ICG fluorescence using a plate reader, and the equivalent amount of fresh buffer was added. For pH 6.0 + L and pH 7.4 + L group, LIPC suspension was irradiated with laser (808 nm, 0.5 W/cm2) for 5 min and the following step was the same as that described above.

### Cellular uptake

CT26, HEK-293T, and RAW 264.7 cells were incubated in DMEM medium containing 10% FBS, 100 U/mL penicillin, and 100 μg/mL streptomycin. All the cells were cultured in a 5% CO_2_ incubator at 37 °C. Cellular internalization of LIPC and LIP was analyzed using LSCM and flow cytometry. CT26 cells were seeded in 24-well plate (1 × 10^5^ cell/well) and cultured overnight. Initially, LIPC with the LDH:CCM mass ratios of 10:1, 20:1, 50:1, 100:1, and 200:1, LIP, and free ICG were added in seeded CT26 cells. After 4 h incubation, the cells were collected, washed with PBS for 3 times for flow cytometry analysis. To prove the specific targeting and immune escaping capability of CCM-LDH NSs (20:1), HEK-293T, B16F0 and RAW 264.7 cells were incubated with LIPC and LIP for 4 h. Afterward, the cells were collected and washed with PBS for flow cytometry analysis.

For LSCM analysis, CT26 cells were seeded in 12-well plate (2 × 10^5^ cell/well) and cultured overnight. Then, Cy3-dsDNA tagged LIPC and LIP containing culture medium was added to the cells. The cells were collected and washed with PBS for 3 times after 4 h incubation, and then fixed with 4% paraformaldehyde, stained with DAPI, and captured by LSCM.

### Cell viability

CT26 cells were seeded in 96-well plate (5000 cells/well) and grown overnight. Next, fresh DMEM medium containing LIPC, LIC, LIP, free ICG, or PTX-BSA (ICG = 0–20 µg/mL, and PTX-BSA = 0–10 µg/mL) was added to replace the previous medium and the cells were continuously cultured for 24 h. After collecting and washing with PBS for 3 times, the cells were dispersed in fresh medium and irradiated with 808 nm laser (0.5 W/cm^2^) for 5 min. Then, the cells were cultured for another 6 h, and their viability was quantified with MTT assay.

### Intracellular ROS detection

ROS generation was investigated by DCFH-DA assay. CT26 cells were seeded in 24-well plate (1 × 10^5^ cell/well) and cultured overnight. After removal of culture medium, the cells were added with fresh medium containing LIPC, LIC and LIP (ICG = 1.2 µg/mL, and PTX-BSA = 0.6 µg/mL) and continuously incubated for 18 h. Then, the cells were washed with PBS for 3 times and replaced by fresh medium. Afterward, the cells were irradiated with 808 nm laser (0.5 W/cm^2^) for 5 min. The cells were cultured for another 6 h, and then added with DCFH-DA (10 µM) and incubated for 20 min. Finally, the fluorescence images were captured in LSCM while the mean fluorescence intensity (MFI) was analyzed based on the flow cytometry data.

### Establishment of tumor model

Female Balb/c mice (6–8 weeks old) were purchased from Biological Resource Facility, the University of Queensland and animal experiments were conducted under the guideline of The University of Queensland Animal Care and Use Committee. The animal tumor model was constructed by subcutaneously injecting CT26 cells (2 × 10^6^ cells, 100 µL) on the right flank of the mouse.

### In vivo homologous targeting and biodistribution of LIPC

When the tumor volume reached appropriately 100 mm^3^, the mice were randomly divided into two groups (n = 5), and intravenously injected with LIPC and LIP (1.2 mg/kg of ICG, 0.6 mg/kg PTX-BSA, and 1.2 mg/kg of CCM), respectively. At 24 h post-injection, the mice were sacrificed and the main organs (heart, liver, spleen, lung, kidney, and tumor) harvested. Fluorescence images of the tumors were captured and MFI of ICG semi-quantified by the IVIS Lumina X5 Imaging system. Meanwhile, fluorescence images of the organs were captured for the analysis of biodistribution.

### In vivo photothermal performance

When the tumor volume reached 50–100 mm^3^, the photothermal performance of LIPC, LIC, and LIP in mice was monitored. The mice were intravenously injected with LIPC, LIC, LIP, and PBS (1.2 mg/kg of ICG, 0.6 mg/kg PTX-BSA, and/or 1.2 mg/kg of CCM), respectively. At 24 h post-injection, the tumors were exposed to 808 nm laser (0.5 W/cm^2^) for 5 min. Photothermal images were captured at 30 s intervals and the temperature was estimated accordingly.

### In vivo ROS detection

CT26 tumor-bearing mice were intravenously injected via tail vein with LIPC, LIC, LIP (1.2 mg/kg of ICG, and/or 0.6 mg/kg PTX-BSA) and PBS. At 24 h post-injection, the mice were intratumorally injected with 50 μL DCFH-DA (100 µM) and irradiated using 808 nm laser (0.5 W/cm^2^) for 5 min. Subsequently, the mice were sacrificed and the tumors harvested for ROS imaging. The MFI of ROS probes was semi-quantified by the IVIS Lumina X5 Imaging system.

### In vivo antitumor efficiency

When the tumor volume reached appropriately 50–100 mm^3^, the mice were randomly divided into 5 groups (n = 5), receiving the treatment of (1) LIPC + L; (2) LIPC; (3) LIC + L; (4) LIP + L; (5) PBS + L. The mice were intravenously injected with LIPC, LIC, LIP (1.2 mg/kg of ICG, and 0.6 mg/kg PTX-BSA), and PBS at day 0 and 7, followed by laser irradiation (0.5 W/cm^2^, 5 min) at day 1 and 8, respectively. The tumor volume and the bodyweight were monitored every other day. After 14 days, the mice were sacrificed and the major organs (heart, liver, spleen, lung, kidney, and tumor) were harvested for H&E staining and TUNEL staining. Tumor volume was calculated with length × width^2^/2.

### Statistical analysis

All the experiments were performed in triplicate with the data presented as mean ± SEM (standard error of the mean) using GraphPad Prism. Student’s *t*-test was used to analyze significant differences. *p*-values less than 0.05 were considered statistically significant. NS: no significant difference when *p* > 0.05, and *p < 0.05, **p < 0.01, ***p < 0.001, and ****p < 0.0001.

## Supplementary Information

**Additional file 1: Fig. S1** Loading efficiency of CCM on LIPC with the different mass ratios of LDH to CCM (10:1, 20:1, 50:1, 100:1 and 200:1). **Fig. S2** The height of **A** LI and **B** LIPC traced along the red line from AFM images. **Fig. S3** Standard curve of ICG. **Fig. S4** Stability of LIPC and LI in PBS at 0, 2, 6, 12 and 24 h measured by DLS. The mass ratio of LDH to CCM was 20:1. **Fig. S5** Stability of LIPC in PBS within 14 days measured by DLS. The mass ratio of LDH to CCM was 20:1. **Fig. S6.** Photothermal performance of LIP under laser irradiation. **A** Temperature change curve and, **B** photothermal images of LIP under laser irradiation (808 nm, 0.5 W/cm^2^) for 5 min. The concentration of ICG was 0, 2.5, 5, 10, and 20 µg/mL. **Fig. S7** Photothermal performance of free ICG under laser irradiation. **A** Temperature change curve and, **B** photothermal images of free ICG under laser irradiation (808 nm, 0.5 W/cm^2^) for 5 min. The concentration of ICG was 0, 2.5, 5, 10 and 20 µg/mL. **Fig. S8** Comparison of photothermal performance in LIP and free ICG group. Temperature change curve of LIP and free ICG under laser irradiation (808 nm, 0.5 W/cm^2^) for 5 min. The concentration of ICG was 5 and 20 µg/mL. **Fig. S9** Photothermal stability of LIPC and free ICG (ICG = 20 µg/mL) under 4 cycles of laser irradiation (808 nm, 0.5 W/cm^2^). **Fig. S10** Drug release profile of ICG from LIPC under different pH and laser irradiation (808 nm, 0.5 W/cm^2^) for 5 min. **Fig. S11** Optimization of the mass ratio of LDH to CCM in LIPC. **A** The MFI and **B** positive cells in CT26 cells after incubation with LIPC with different mass ratios of LDH to CCM (10:1- 200:1), LIP and free ICG for 4 h by flow cytometry. The concentration of ICG was 0.5 µg/mL. **Fig. S12** Positive cells in CT26 cells treated with LIPC for 4 h, laser irradiation (808 nm, 0.5 W/cm^2^) for 5 min and incubated for another 1 h. The concentration of ICG was 0.5 µg/mL. **Fig. S13** Positive cells in RAW 264.7 cells after incubation with LIPC and LIP for 4 h. The concentration of ICG was 0.5 µg/mL. **Fig. S14 A** The MFI and **B-C** positive cells in B16F0 cells after incubation with LIPC and LIP for 4 h. The concentration of ICG was 0.5 µg/mL. **Fig. S15 A** The MFI and **B-C** positive cells in HEK-293 T cells after incubation with LIPC and LIP for 4 h. The concentration of ICG was 0.5 µg/mL. **Fig. S16 A** The MFI and **B-C** positive cells in CT26 cells after incubation with Cy3-dsDNA tagged LIPC and LIP for 4 h, laser irradiation (808 nm, 0.5 W/cm^2^) for 5 min and incubation for another 1 h. The concentration of Cy3-dsDNA was 40 nM. **Fig. S17** Cell viability of CT26 cells treated with LIC, LI, and free ICG in the dark. **Fig. S18** The production of ROS in CT26 cells treated with LIPC, LIC, LIP and LI under laser irradiation (808 nm, 0.5 W/cm^2^) for 5 min. **Fig. S19** The temperature of mice treated with LIPC + L (808 nm, 0.5 W/cm2) at 5 min **B** traced along the white line **A**. **Fig. S20** In vivo photothermal performance of LIPC. **A** Temperature change curve and, **B** photothermal images of the mice under laser irradiation (808 nm, 0.3, 0.5 and 0.7 W/cm^2^) for 5 min. **Fig. S21** H&E staining of the organs in mice after different treatments. Scale bar: 200 µm. **Table. S1** Size, zeta potential and PDI of LIPC with different mass ratios of LDH to CCM. **Table. S2** Synergistic effects of photo-chemotherapy (C) by combing phototherapy (A) and chemotherapy (B).

## Data Availability

Without restrictions.
