# Supplementary material for: Biomimetic 2D layered double hydroxide nanocomposites for hyperthermia-facilitated homologous targeting cancer photo-chemotherapy
Source: J Nanobiotechnology. 2021 Oct 30;19:351. doi: 10.1186/s12951-021-01096-9 (PMC8557519; doi:10.1186/s12951-021-01096-9)
Supplement: Supplementary file 1 — Additional file 1: Fig. S1 Loading efficiency of CCM on LIPC with the different mass ratios of LDH to CCM (10:1, 20:1, 50:1, 100:1 and 200:1). Fig. S2 The height of A LI and B LIPC traced along the red line from AFM images. Fig. S3 Standard curve of ICG. Fig. S4 Stability of LIPC and LI in PBS at 0, 2, 6, 12 and 24 h measured by DLS. The mass ratio of LDH to CCM was 20:1. Fig. S5 Stability of LIPC in PBS within 14 days measured by DLS. The mass ratio of LDH to CCM was 20:1. Fig. S6. Photothermal performance of LIP under laser irradiation. A Temperature change curve and, B photothermal images of LIP under laser irradiation (808 nm, 0.5 W/cm2) for 5 min. The concentration of ICG was 0, 2.5, 5, 10, and 20 µg/mL. Fig. S7 Photothermal performance of free ICG under laser irradiation. A Temperature change curve and, B photothermal images of free ICG under laser irradiation (808 nm, 0.5 W/cm2) for 5 min. The concentration of ICG was 0, 2.5, 5, 10 and 20 µg/mL. Fig. S8 Comparison of photothermal performance in LIP and free ICG group. Temperature change curve of LIP and free ICG under laser irradiation (808 nm, 0.5 W/cm2) for 5 min. The concentration of ICG was 5 and 20 µg/mL. Fig. S9 Photothermal stability of LIPC and free ICG (ICG = 20 µg/mL) under 4 cycles of laser irradiation (808 nm, 0.5 W/cm2). Fig. S10 Drug release profile of ICG from LIPC under different pH and laser irradiation (808 nm, 0.5 W/cm2) for 5 min. Fig. S11 Optimization of the mass ratio of LDH to CCM in LIPC. A The MFI and B positive cells in CT26 cells after incubation with LIPC with different mass ratios of LDH to CCM (10:1- 200:1), LIP and free ICG for 4 h by flow cytometry. The concentration of ICG was 0.5 µg/mL. Fig. S12 Positive cells in CT26 cells treated with LIPC for 4 h, laser irradiation (808 nm, 0.5 W/cm2) for 5 min and incubated for another 1 h. The concentration of ICG was 0.5 µg/mL. Fig. S13 Positive cells in RAW 264.7 cells after incubation with LIPC and LIP for 4 h. The concentration of IC [file 12951_2021_1096_MOESM1_ESM.docx]

Supplementary Information

**Biomimetic 2D layered double hydroxide nanocomposites for hyperthermia-facilitated homologous targeting cancer photo-chemotherapy**

Jingjing Wang, Luyao Sun, Jie Liu, Bing Sun, Li Li* and Zhi Ping Xu*

*Australian Institute for Bioengineering and Nanotechnology, The University of Queensland, Brisbane, QLD 4072, Australia*

** Corresponding authors.*

*E-mail:* [*gordonxu@uq.edu.au*](mailto:gordonxu@uq.edu.au) *and* [*l.li2@uq.edu.au*](mailto:l.li2@uq.edu.au)

1. **Supplementary Figures S1-S21**
2. **Supplementary Tables S1-S2**

**Supplementary Figures**


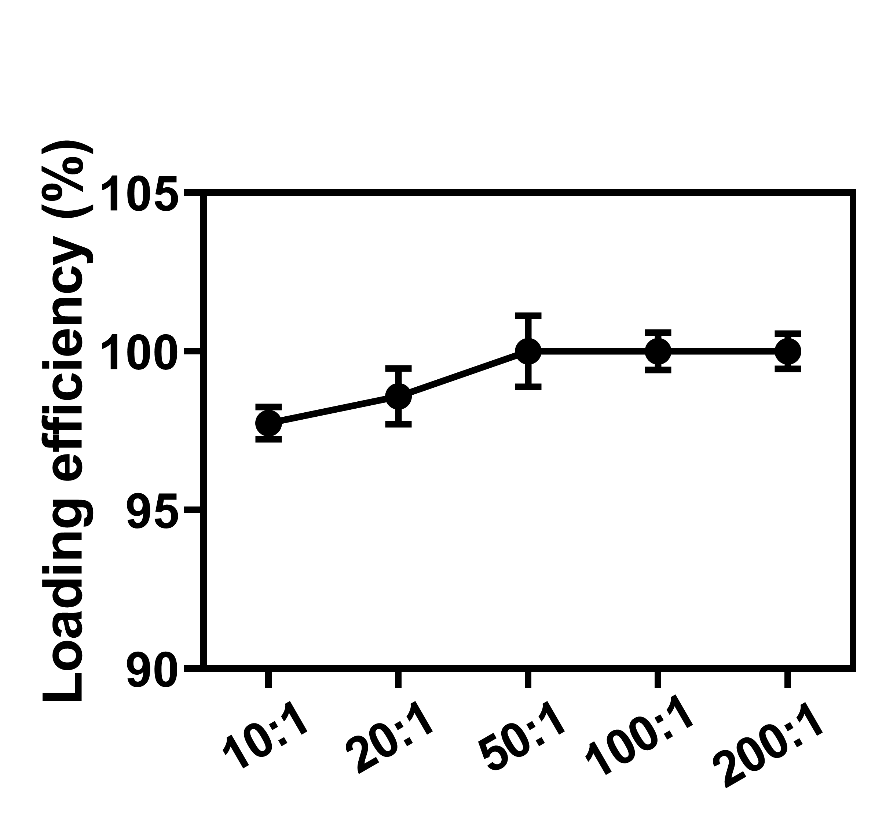


**Fig. S1** Loading efficiency of CCM on LIPC with the different mass ratios of LDH to CCM (10:1, 20:1, 50:1, 100:1 and 200:1).


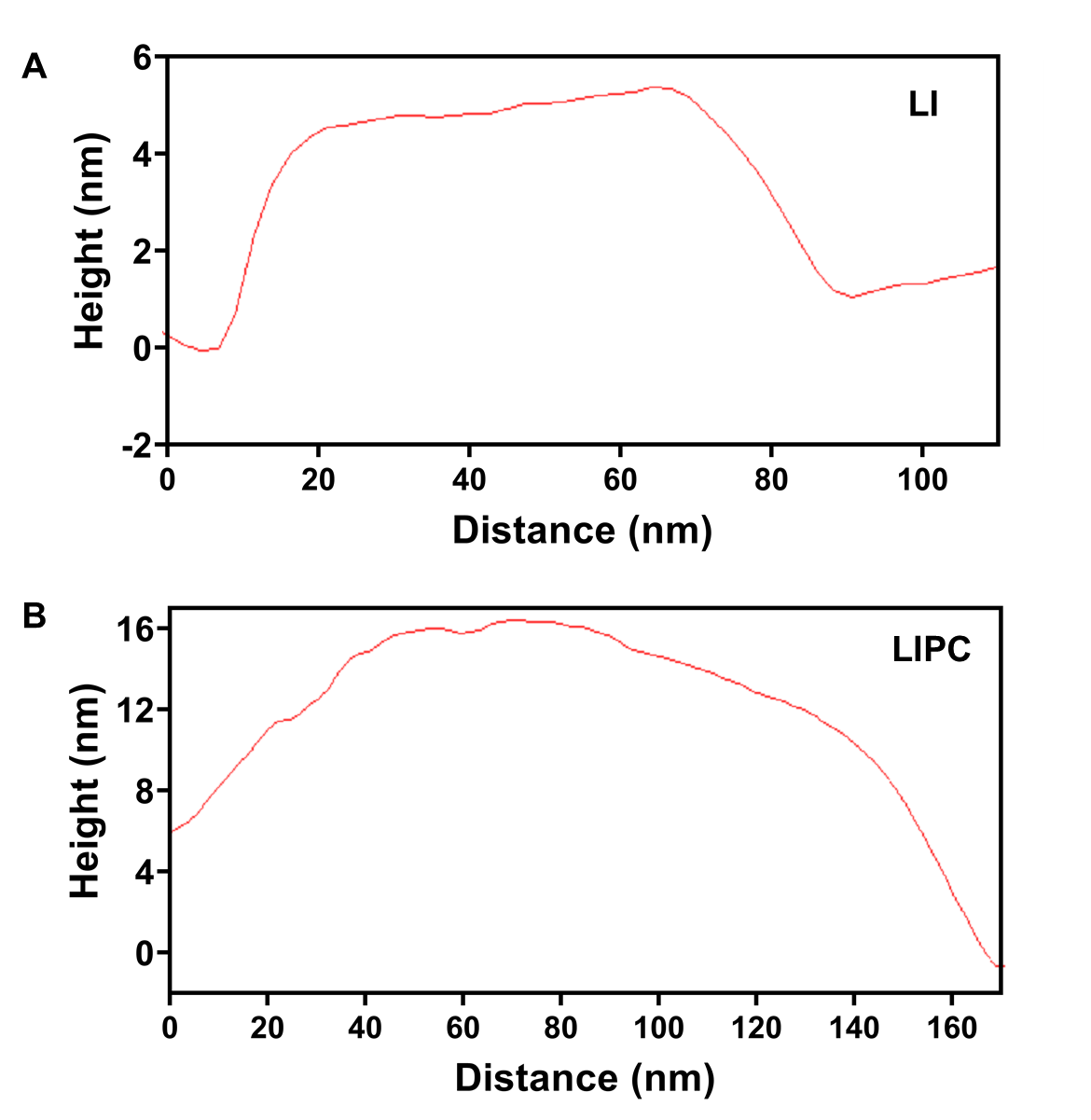


**Fig. S2** The height of **A** LI and **B** LIPC traced along the red line from AFM images.


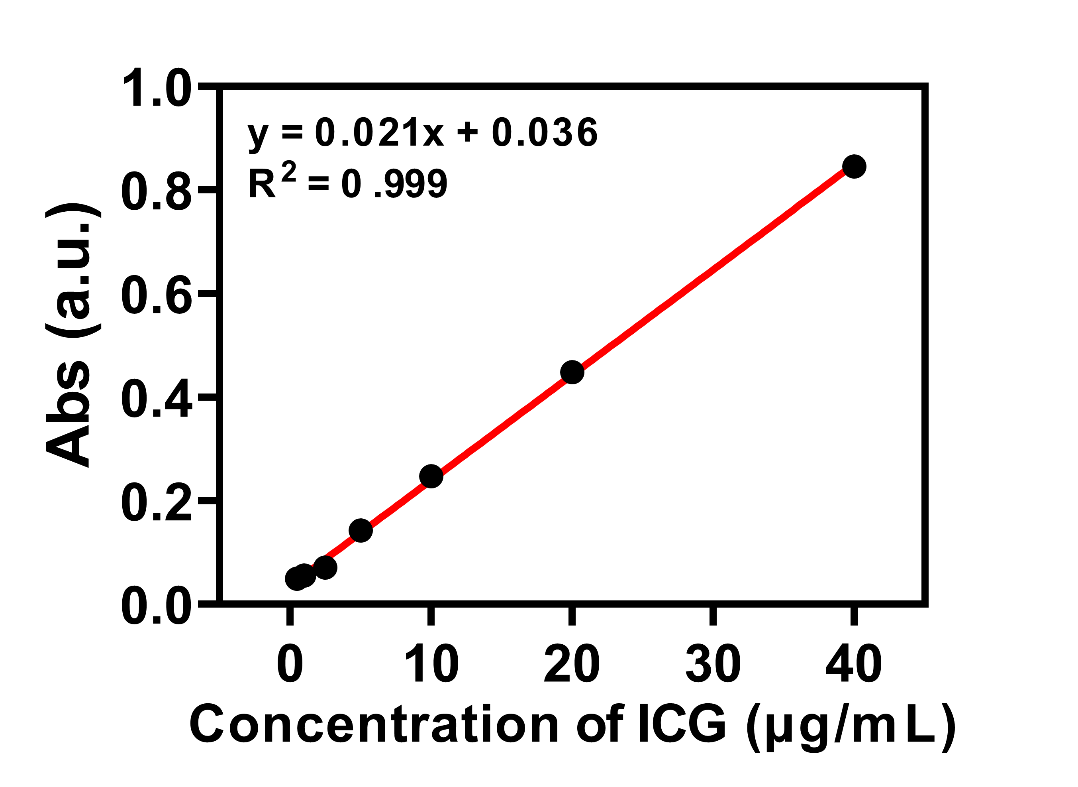


**Fig. S3** Standard curve of ICG.


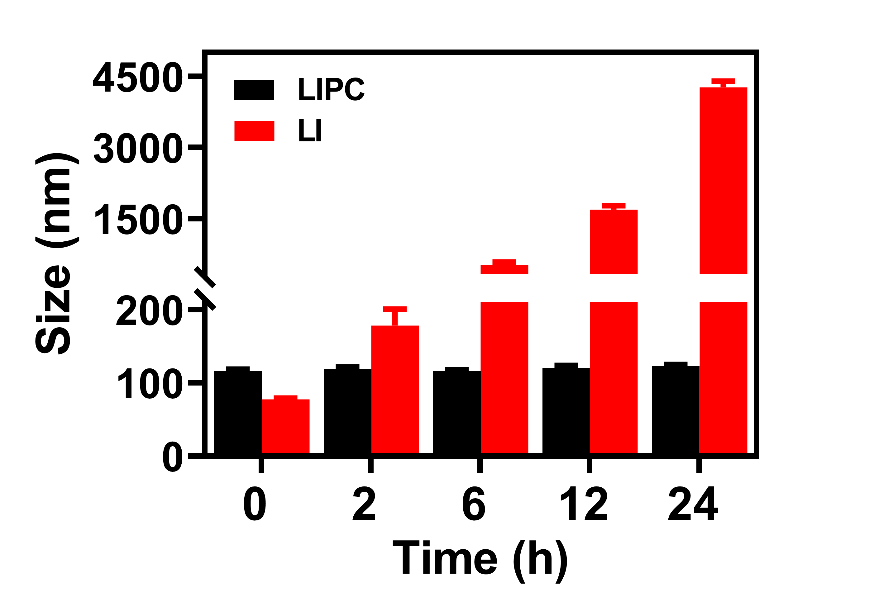


**Fig. S4** Stability of LIPC and LI in PBS at 0, 2, 6, 12 and 24 h measured by DLS. The mass ratio of LDH to CCM was 20:1.


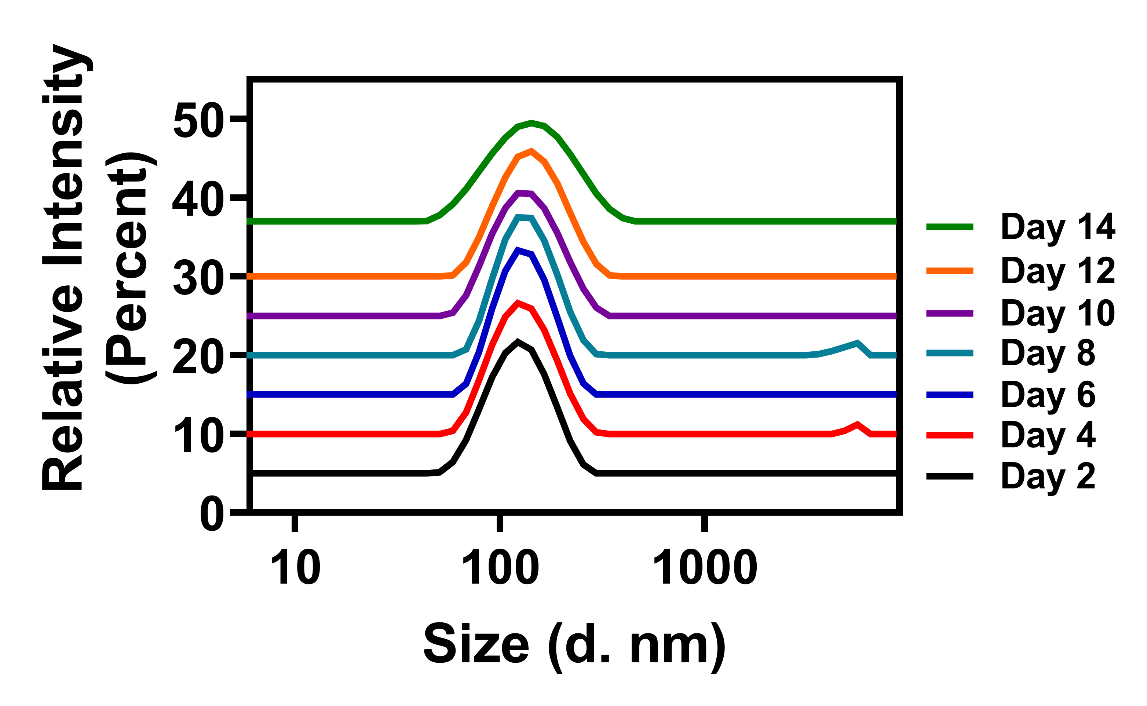


**Fig. S5** Stability of LIPC in PBS within 14 days measured by DLS. The mass ratio of LDH to CCM was 20:1.


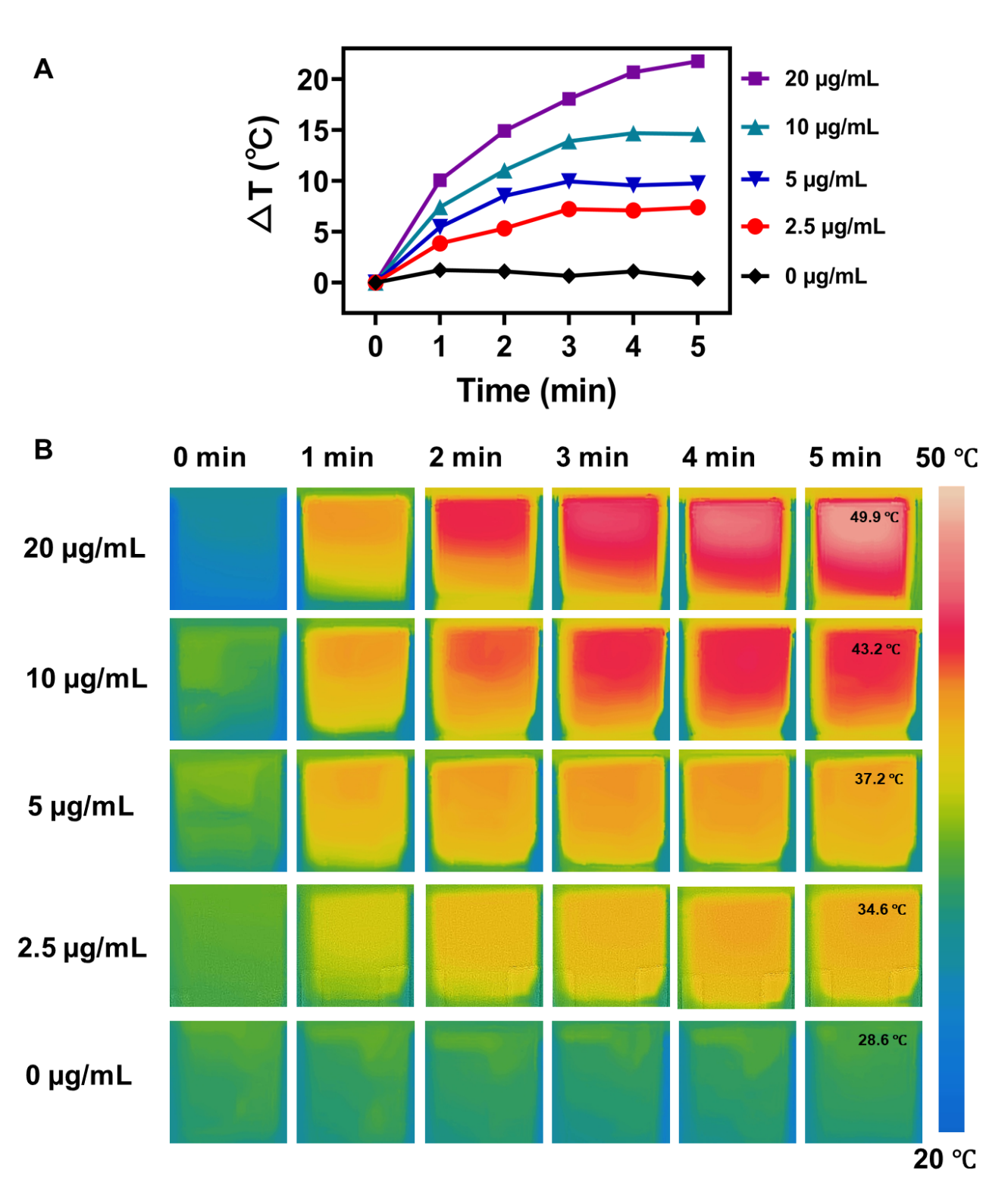


**Fig. S6.** Photothermal performance of LIP under laser irradiation. **A** Temperature change curve and, **B** photothermal images of LIP under laser irradiation (808 nm, 0.5 W/cm^2^) for 5 min. The concentration of ICG was 0, 2.5, 5, 10, and 20 µg/mL.


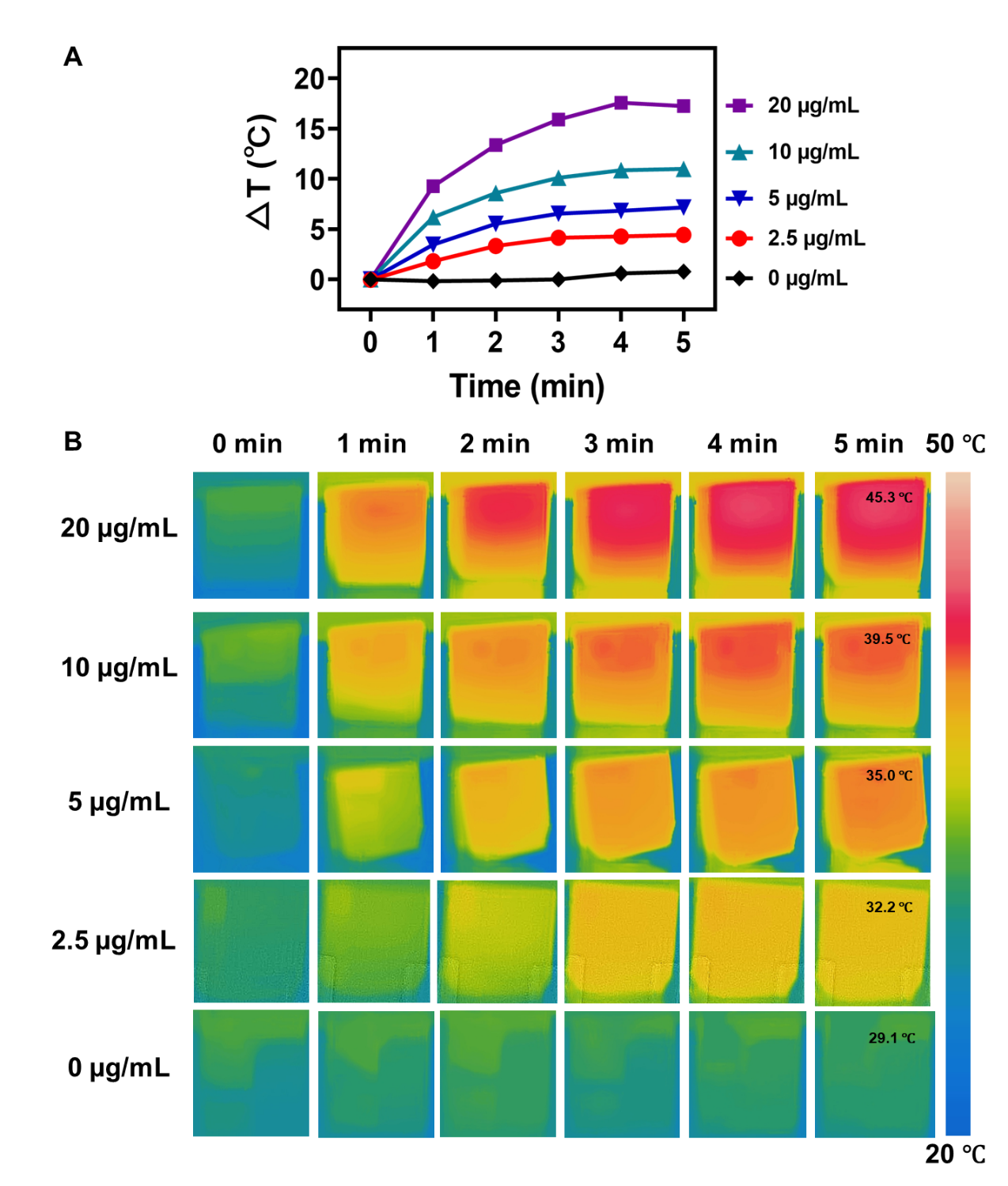


**Fig. S7** Photothermal performance of free ICG under laser irradiation. **A** Temperature change curve and, **B** photothermal images of free ICG under laser irradiation (808 nm, 0.5 W/cm^2^) for 5 min. The concentration of ICG was 0, 2.5, 5, 10 and 20 µg/mL.


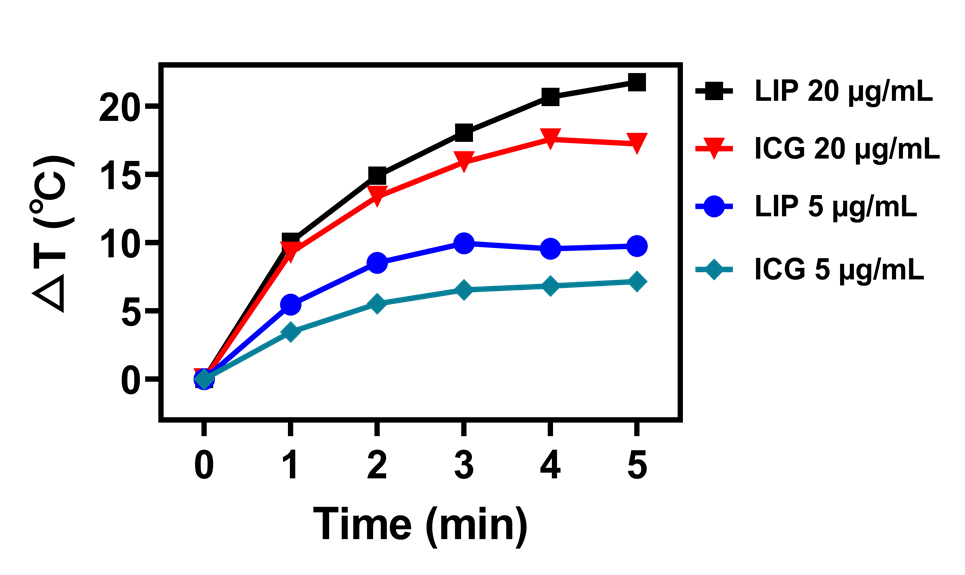


**Fig. S8** Comparison of photothermal performance in LIP and free ICG group. Temperature change curve of LIP and free ICG under laser irradiation (808 nm, 0.5 W/cm^2^) for 5 min. The concentration of ICG was 5 and 20 µg/mL.


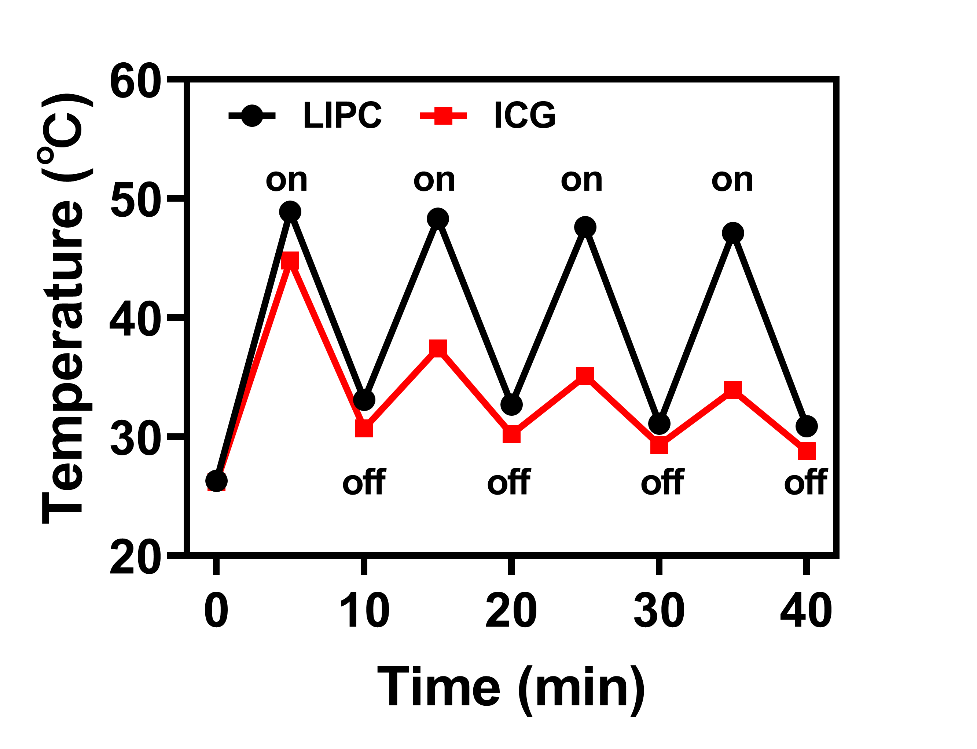


**Fig. S9** Photothermal stability of LIPC and free ICG (ICG = 20 µg/mL) under 4 cycles of laser irradiation (808 nm, 0.5 W/cm^2^).


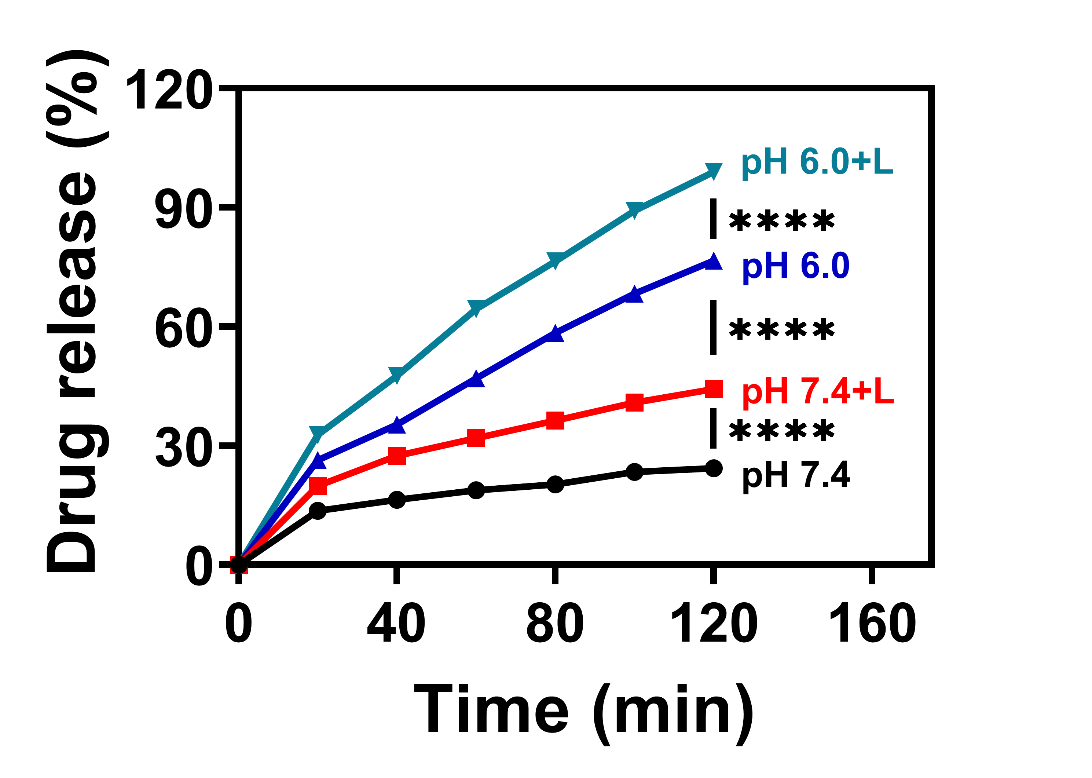


**Fig. S10** Drug release profile of ICG from LIPC under different pH and laser irradiation (808 nm, 0.5 W/cm^2^) for 5 min.


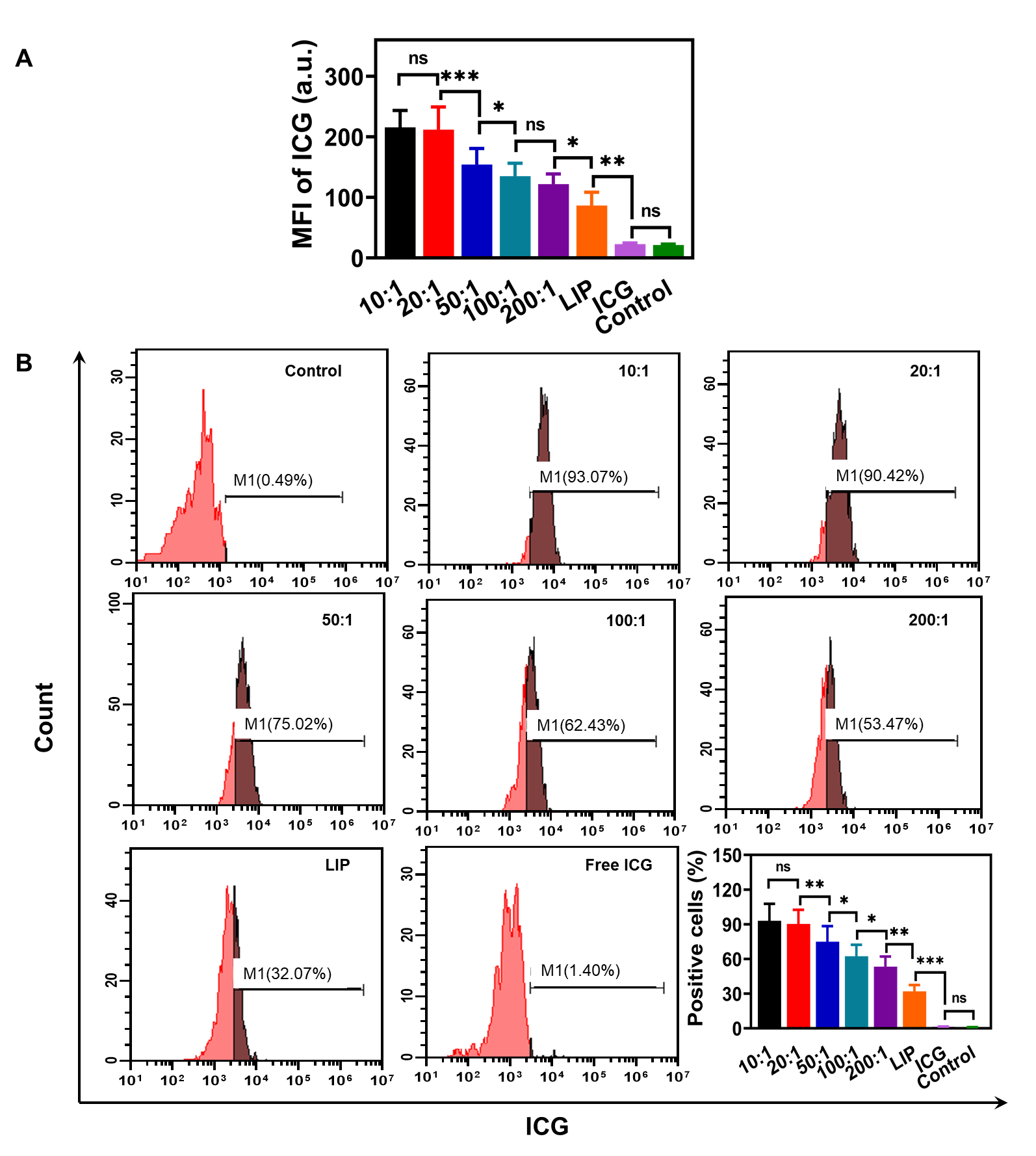


**Fig. S11** Optimization of the mass ratio of LDH to CCM in LIPC. **A** The MFI and **B** positive cells in CT26 cells after incubation with LIPC with different mass ratios of LDH to CCM (10:1- 200:1), LIP and free ICG for 4 h by flow cytometry. The concentration of ICG was 0.5 µg/mL.


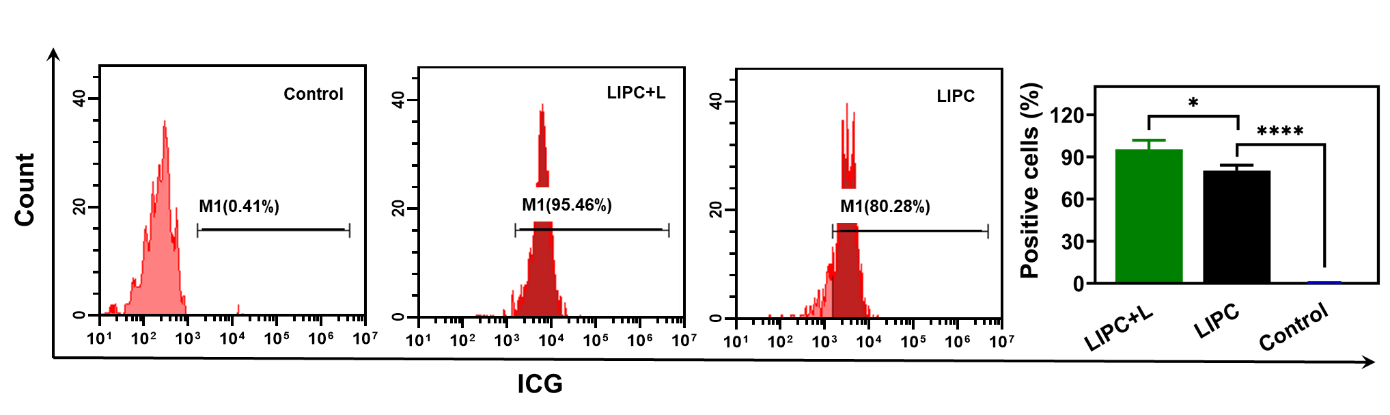


**Fig. S12** Positive cells in CT26 cells treated with LIPC for 4 h, laser irradiation (808 nm, 0.5 W/cm^2^) for 5 min and incubated for another 1 h. The concentration of ICG was 0.5 µg/mL.


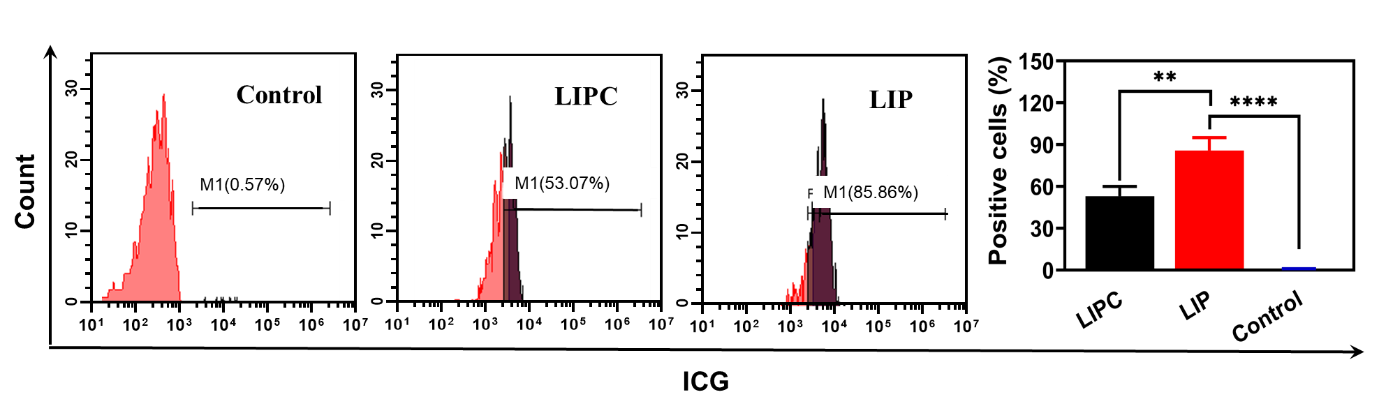


**Fig. S13** Positive cells in RAW 264.7 cells after incubation with LIPC and LIP for 4 h. The concentration of ICG was 0.5 µg/mL.


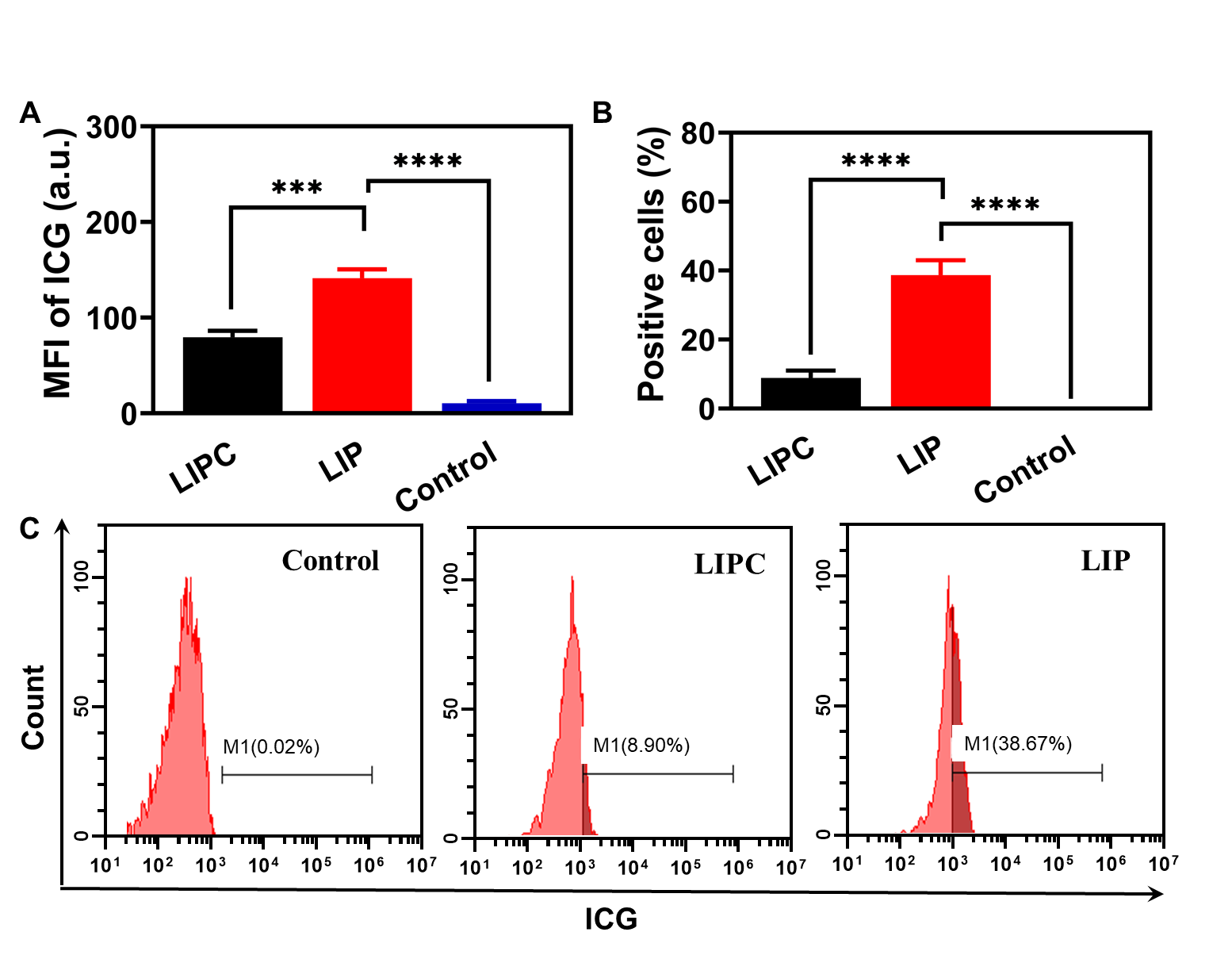


**Fig. S14** **A** The MFI and **B-C** positive cells in B16F0 cells after incubation with LIPC and LIP for 4 h. The concentration of ICG was 0.5 µg/mL.


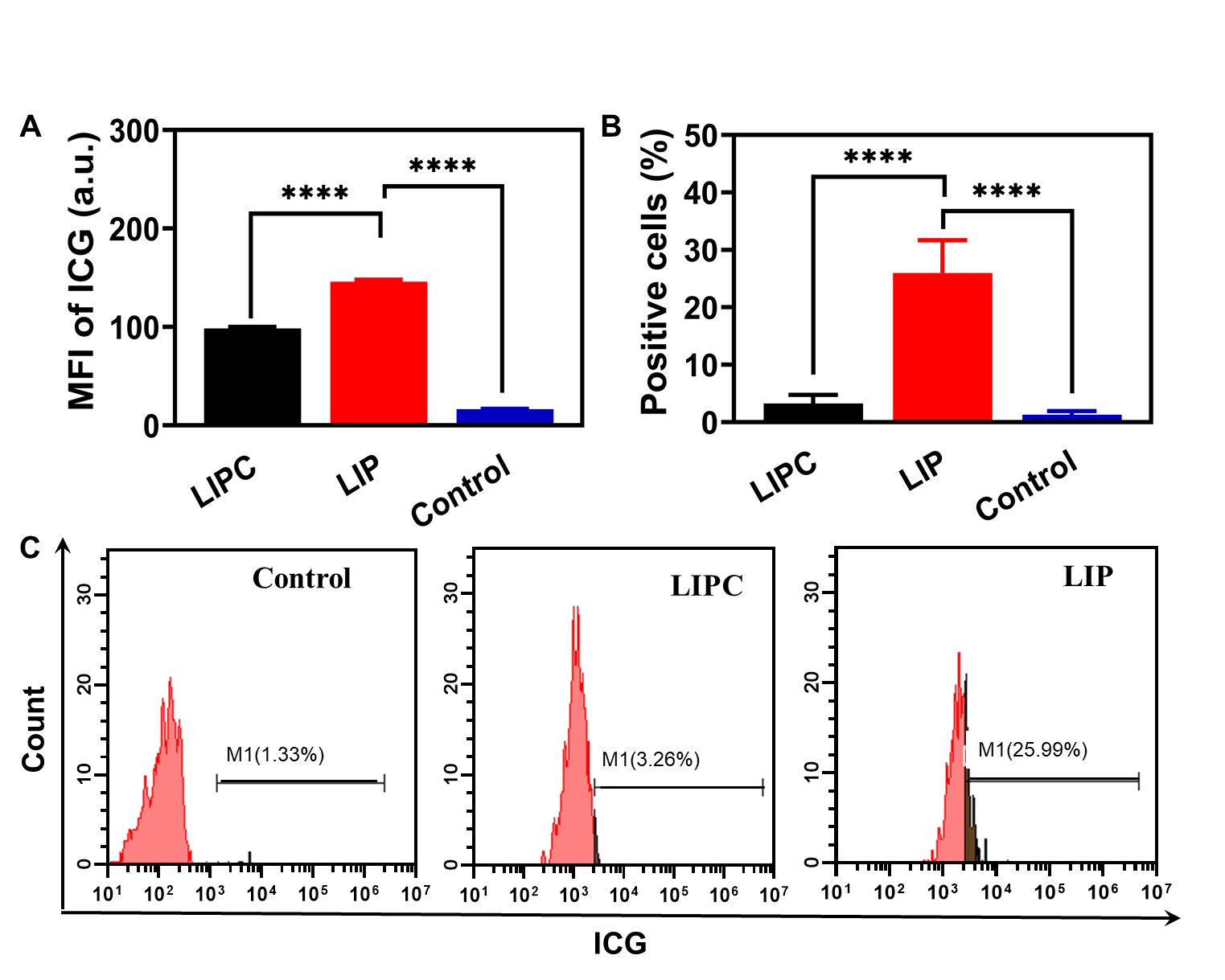


**Fig. S15** **A** The MFI and **B-C** positive cells in HEK-293T cells after incubation with LIPC and LIP for 4 h. The concentration of ICG was 0.5 µg/mL.


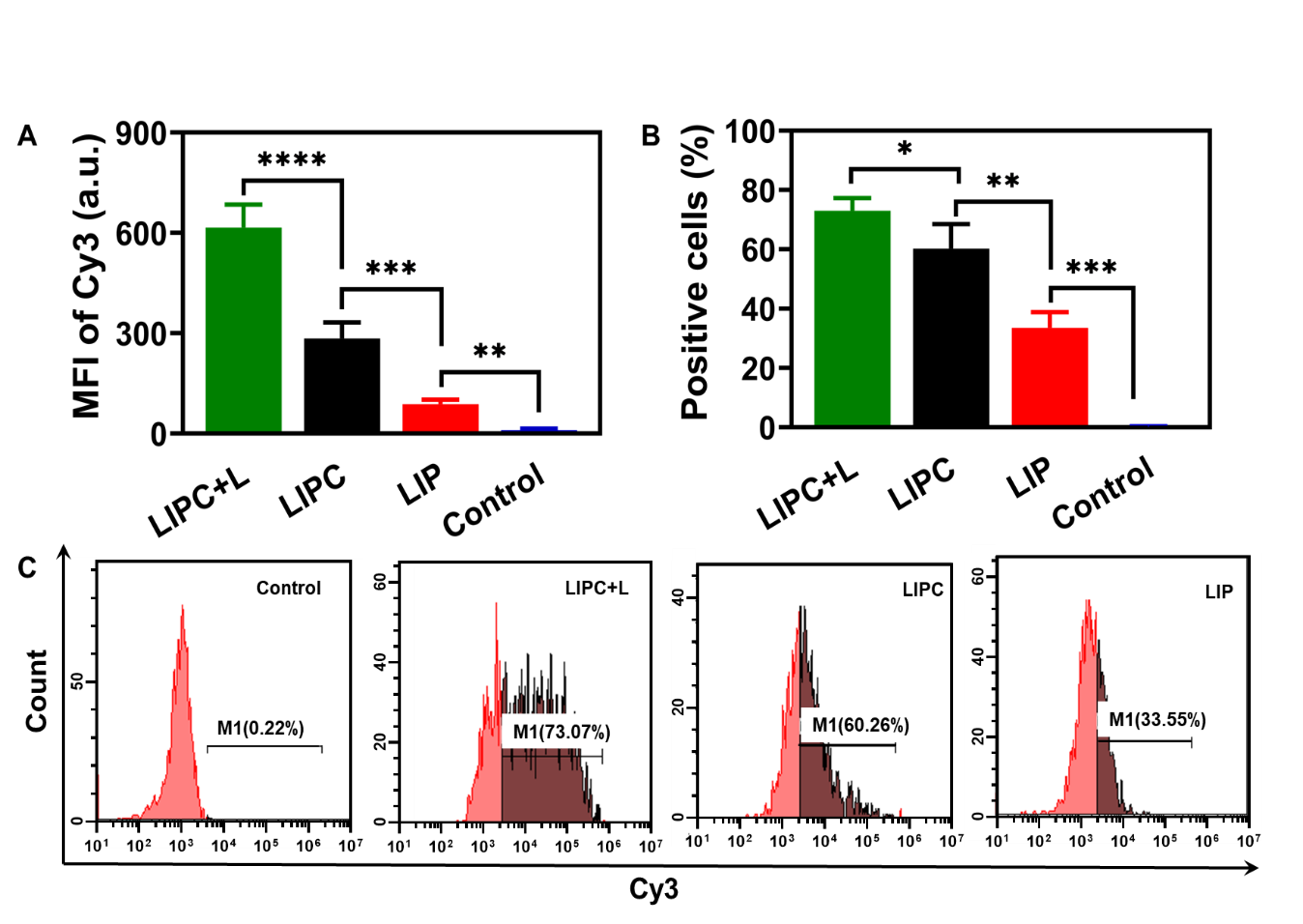


**Fig. S16** **A** The MFI and **B-C** positive cells in CT26 cells after incubation with Cy3-dsDNA tagged LIPC and LIP for 4 h, laser irradiation (808 nm, 0.5 W/cm^2^) for 5 min and incubation for another 1 h. The concentration of Cy3-dsDNA was 40 nM.


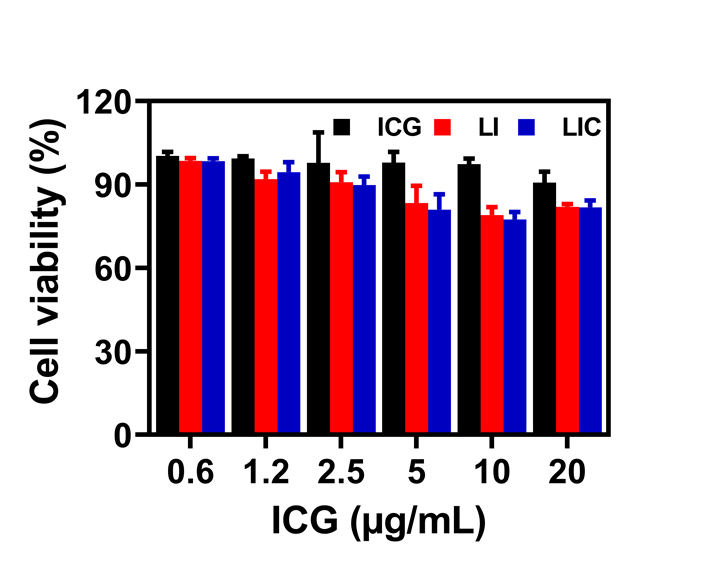


**Fig. S17** Cell viability of CT26 cells treated with LIC, LI, and free ICG in the dark.


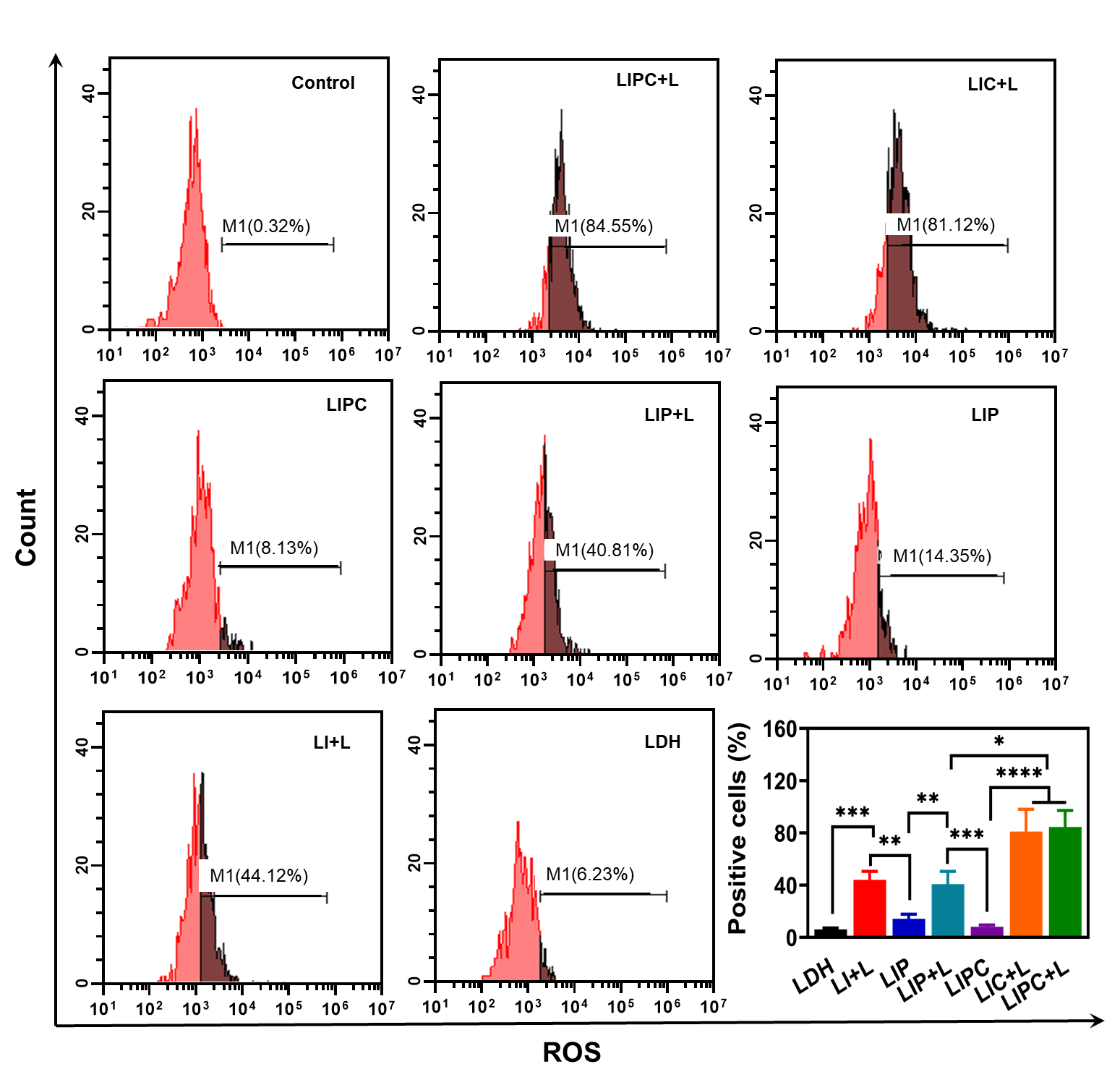


**Fig. S18** The production of ROS in CT26 cells treated with LIPC, LIC, LIP and LI under laser irradiation (808 nm, 0.5 W/cm^2^) for 5 min.


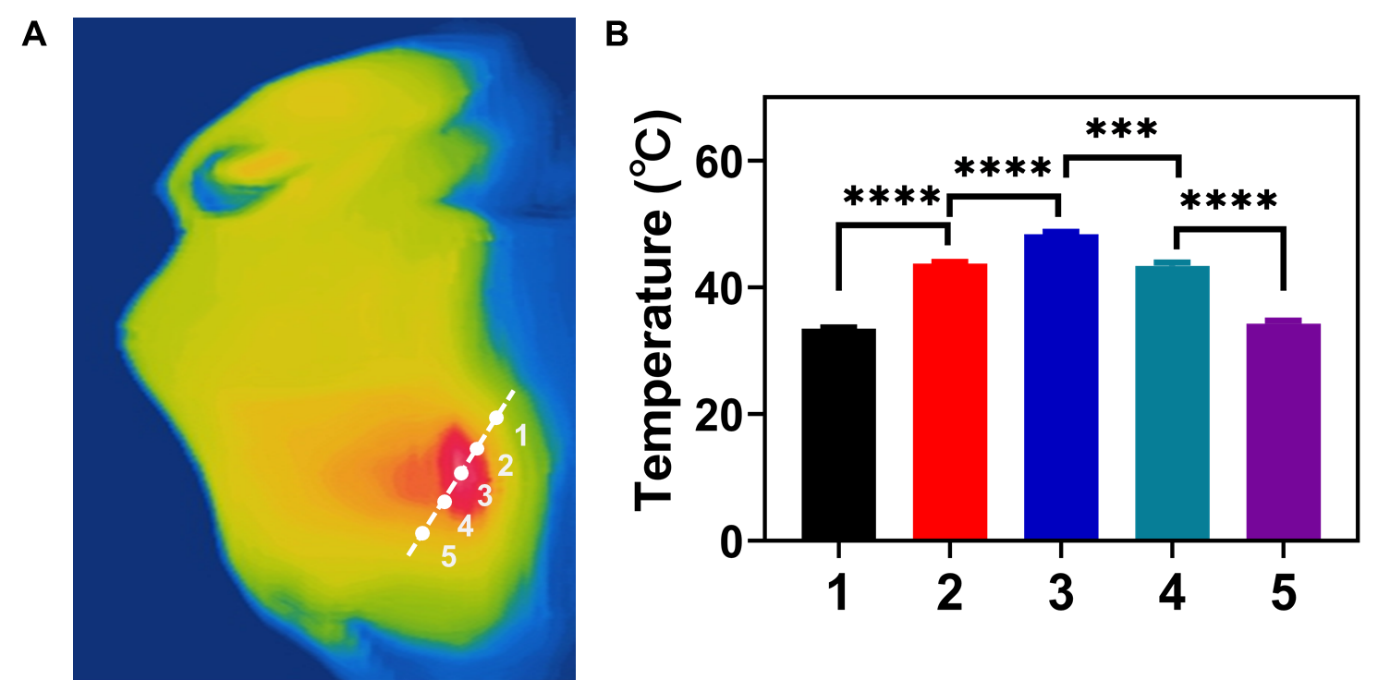


**Fig. S19** The temperature of mice treated with LIPC+L (808 nm, 0.5 W/cm^2^) at 5 min **B** traced along the white line **A**.


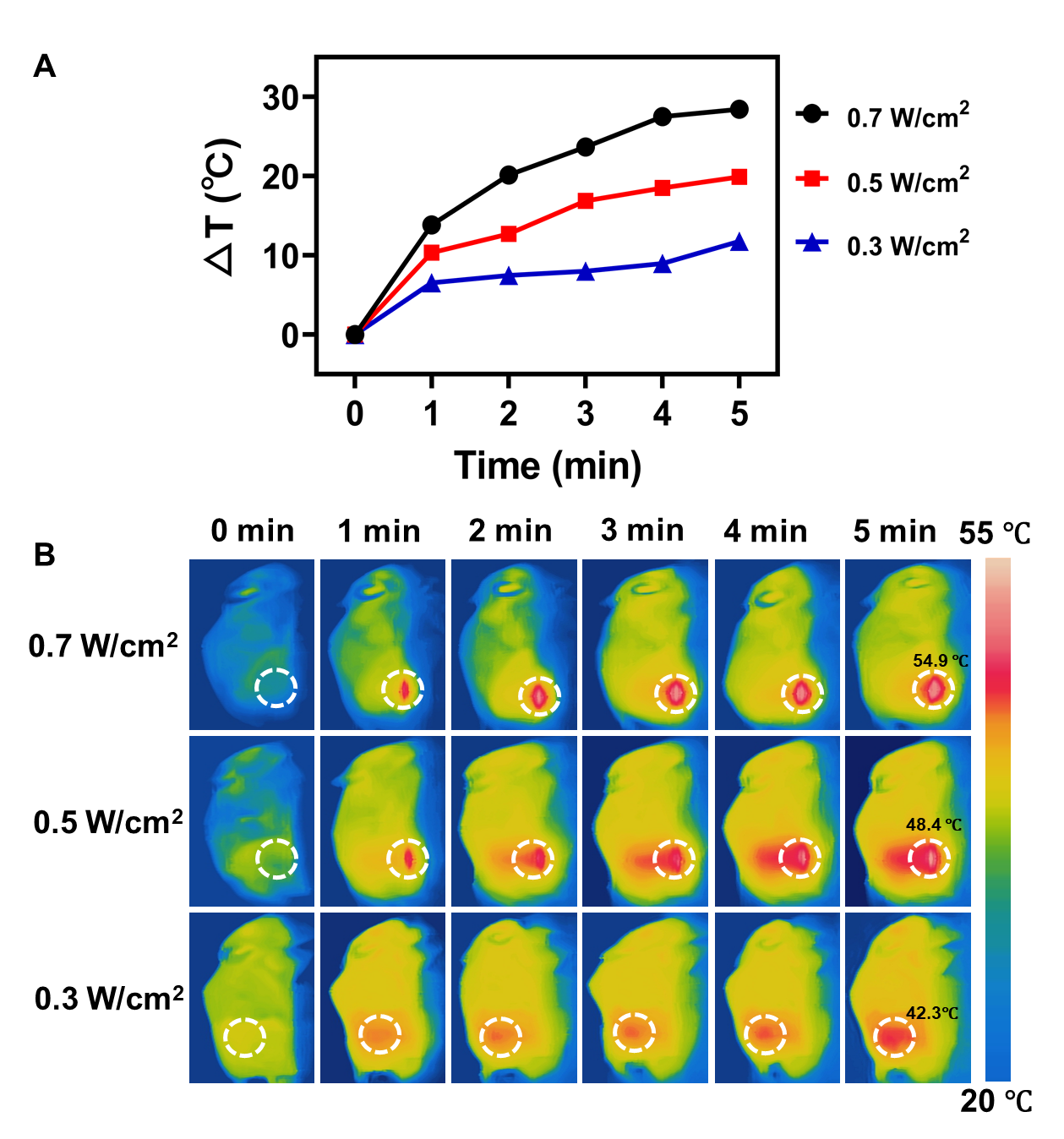


**Fig. S20** *In vivo* photothermal performance of LIPC. **A** Temperature change curve and, **B** photothermal images of the mice under laser irradiation (808 nm, 0.3, 0.5 and 0.7 W/cm^2^) for 5 min.


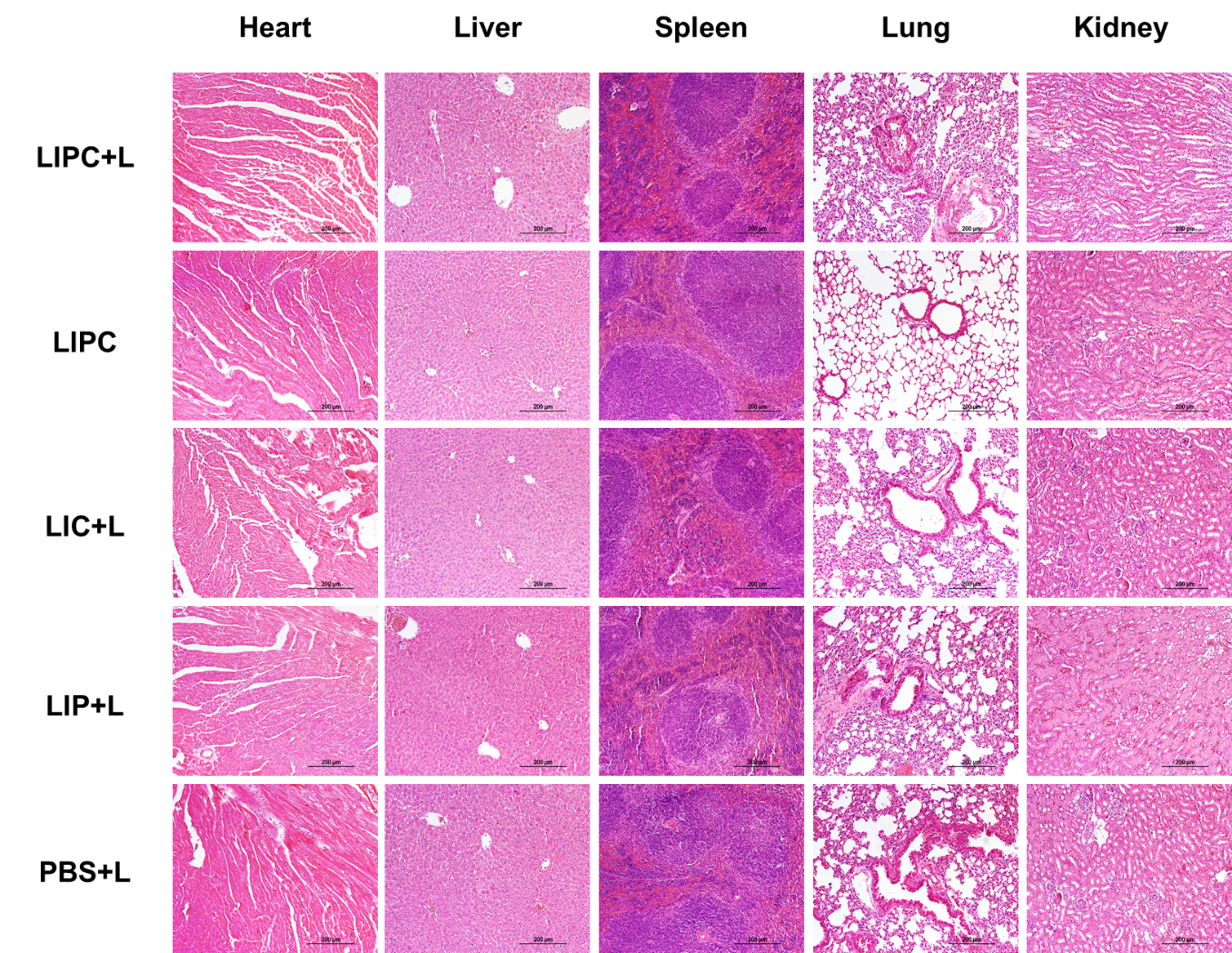


**Fig. S21** H&E staining of the organs in mice after different treatments. Scale bar: 200 µm.

**Table. S1** Size, zeta potential and PDI of LIPC with different mass ratios of LDH to CCM.

| LDH:CCM | 10:1 | 20:1 | 50:1 | 100:1 | 200:1 |
| --- | --- | --- | --- | --- | --- |
| Size (d. nm) | 120.2 ± 5.5 | 116.3 ± 4.5 | 114.2 ± 4.9 | 115.6 ± 3.4 | 113.8 ± 2.3 |
| Zeta potential (mV) | -20.4 ± 4.1 | -19.2 ± 2.7 | -16.5 ± 3.7 | -15.9 ± 1.8 | -15.1 ± 3.4 |
| PDI | 0.283 | 0.271 | 0.216 | 0.214 | 0.227 |

**Table. S2** Synergistic effects of photo-chemotherapy (C) by combing phototherapy (A) and chemotherapy (B).

| Phototherapy (A) | Chemotherapy (B) | Photo-chemotherapy (C) | Combination index (A×B/C) | Synergy |
| --- | --- | --- | --- | --- |
| LI+L (74.2%) | LIP (69.8%) | LIP+L (43.3%) | 1.20 | mild |
| LIC+L (46.3%) | LIPC (44.7%) | LIPC+L (16.3%) | 1.27 | mild |

The concentration of ICG, PTX-BSA, and CCM were 1.2, 0.6, and 2.5 µg/mL, respectively

A*B/C ratio (CI):

<0.8: asynergy

0.8-1.2: additive

1.2-1.4: mild synergy

1.4-1.6: moderate synergy

>1.6: strong synergy
